# Identification of novel genes involved in apoptosis of HIV-infected macrophages using unbiased genome-wide screening

**DOI:** 10.1186/s12879-021-06346-7

**Published:** 2021-07-07

**Authors:** Simon X. M. Dong, Frederick S. Vizeacoumar, Kalpana K. Bhanumathy, Nezeka Alli, Cristina Gonzalez-Lopez, Niranjala Gajanayaka, Ramon Caballero, Hamza Ali, Andrew Freywald, Edana Cassol, Jonathan B. Angel, Franco J. Vizeacoumar, Ashok Kumar

**Affiliations:** 1 Apoptosis Research Center, Children’s Hospital of Eastern Ontario, Faculty of Medicine, University of Ottawa, Ottawa, ON Canada; 2 Department of Microbiology and Immunology, Faculty of Medicine, University of Ottawa, Ottawa, ON Canada; 3 Department of Pathology, College of Medicine, University of Saskatchewan, Saskatoon, SK Canada; 4 Cancer Research, Saskatchewan Cancer Agency, 107 Wiggins Road, Saskatoon, SK Canada; 5 Department of Health Sciences, Carleton University, Ottawa, ON Canada; 6 Department of Medicine, the Ottawa Health Research Institute, Faculty of Medicine, University of Ottawa, Ottawa, ON Canada; 7 Department of Pathology and Laboratory Medicine, Faculty of Medicine, University of Ottawa, Ottawa, ON Canada

**Keywords:** AIDS, Apoptosis, HIV reservoir, Genome-wide screening, Selective killing, Macrophages, 90K lentivirus shRNA pool technology

## Abstract

**Background:**

Macrophages, besides resting latently infected CD4+ T cells, constitute the predominant stable, major non-T cell HIV reservoirs. Therefore, it is essential to eliminate both latently infected CD4+ T cells and tissue macrophages to completely eradicate HIV in patients. Until now, most of the research focus is directed towards eliminating latently infected CD4+ T cells. However, few approaches have been directed at killing of HIV-infected macrophages either in vitro or in vivo*.* HIV infection dysregulates the expression of many host genes essential for the survival of infected cells. We postulated that exploiting this alteration may yield novel targets for the selective killing of infected macrophages.

**Methods:**

We applied a pooled shRNA-based genome-wide approach by employing a lentivirus-based library of shRNAs to screen novel gene targets whose inhibition should selectively induce apoptosis in HIV-infected macrophages. Primary human MDMs were infected with HIV-eGFP and HIV-HSA viruses. Infected MDMs were transfected with siRNAs specific for the promising genes followed by analysis of apoptosis by flow cytometry using labelled Annexin-V in HIV-infected, HIV-exposed but uninfected bystander MDMs and uninfected MDMs. The results were analyzed using student’s t-test from at least four independent experiments.

**Results:**

We validated 28 top hits in two independent HIV infection models. This culminated in the identification of four target genes, *Cox7a2*, *Znf484*, *Cstf2t*, and *Cdk2*, whose loss-of-function induced apoptosis preferentially in HIV-infected macrophages. Silencing these single genes killed significantly higher number of HIV-HSA-infected MDMs compared to the HIV-HSA-exposed, uninfected bystander macrophages, indicating the specificity in the killing of HIV-infected macrophages. The mechanism governing *Cox7a2-*mediated apoptosis of HIV-infected macrophages revealed that targeting respiratory chain complex II and IV genes also selectively induced apoptosis of HIV-infected macrophages possibly through enhanced ROS production.

**Conclusions:**

We have identified above-mentioned novel genes and specifically the respiratory chain complex II and IV genes whose silencing may cause selective elimination of HIV-infected macrophages and eventually the HIV-macrophage reservoirs. The results highlight the potential of the identified genes as targets for eliminating HIV-infected macrophages in physiological environment as part of an HIV cure strategy.

**Supplementary Information:**

The online version contains supplementary material available at 10.1186/s12879-021-06346-7.

## Background

Human immunodeficiency virus (HIV) persists in infected individuals and remains as a lifelong infection even after prolonged periods on the suppressive anti-retroviral therapy (ART) because of the establishment of HIV reservoirs [1, 2]. ART interruption is accompanied by rebound viremia, primarily following activation of latently infected CD4+ T cells and infected macrophages [3]. Although resting latently infected CD4+ T cells constitute the predominant HIV reservoirs, it has been established that macrophages serve as one of the major non-T cell reservoir [4]. Macrophages are the early targets for HIV and are productively infected [5, 6]. Unlike CD4+ T cells which are characterized by a fast and irreversible depletion [7], infected macrophages survive active viral replication with a half-life ranging from months to years, respond poorly to ART [8, 9], develop resistance to apoptosis and HIV cytopathic effects, and harbour unintegrated and integrated viral DNA in a state of latency [10, 11]. In patients undergoing effective ART, infected macrophages shield HIV viruses against a spectrum of host anti-viral responses [12, 13]. Moreover, HIV is retained in the infected macrophages within unique virus containing compartments wherein the virions are protected from neutralizing antibodies and anti-viral drugs [14, 15]. Recent evidence has suggested that tissue resident macrophages homing in lungs, liver, spleen and brain, are derived from embryonic yolk sac progenitor cells and are capable of self-renewal with little to no contribution from the circulating monocytes during homeostasis [16]. These resident macrophages remain as a long-term HIV-infected macrophage pools in the tissues without being superseded by the blood monocyte-derived macrophages [17], thereby serving as stable viral reservoirs. Therefore, it is essential to eliminate both latently infected CD4+ T cells and tissue macrophages to completely eradicate HIV in patients subjected to ART [6].

Over the last several years, most of the research focus is directed towards eliminating latently infected CD4+ T cells through a shock and kill approach by employing strategies to reactivate HIV and eliminate reactivated cells by host immunity [18, 19]. However, few approaches have been focussed on killing of HIV-infected macrophages either in vitro or in vivo*.* For example, galactin-3 [20], motexafin gadolinium [21], TNF Related Apoptosis Inducing Ligand (TRAIL) [22], and colony-stimulating factor 1 receptor antagonists [23] have been shown to induce apoptotic cell death in HIV-infected macrophages with limited success. We and others have shown that HIV infection dysregulates the expression of many host genes essential for the survival of infected cells [24, 25], suggesting that targeting genes required for cell survival specifically at this altered molecular context may selectively induce apoptosis in HIV-infected macrophages. We postulated that exploiting this alteration may yield novel targets for the selective killing of infected macrophages and ultimately lead to the development of treatments that can serve as part of a HIV cure strategy. As loss-of-function screens are being increasingly applied to understand disease mechanisms [26], we performed a genome-wide screen by employing a lentivirus-based library of shRNAs to identify novel gene targets, whose inhibition should selectively induce apoptosis in HIV-infected macrophages. Herein, we report the screening of ~ 18,000 genes, and subsequent validation of 28 top hits in two viral models to identify four potential target genes, *Cox7a2*, *Znf484*, *Cstf2t*, and *Cdk2*, whose loss-of-function induced apoptosis of HIV-infected macrophages. The mechanisms governing *Cox7a2-*associated apoptosis revealed that suppression of respiratory chain complexes II and IV genes can selectively induce apoptosis in HIV-infected macrophages.

## Methods

### Cell culture and differentiation of U937 and THP-1 cells and preparation of primary MDMs

U937, a pro-monocytic, human myeloid leukaemia cell line, and THP-1 cells, a human monocytic cell line derived from an acute monocytic leukemia patient, were purchased from ATCC (Cat. CRL-1593.2™, and Cat. TIB-202™). Cells were maintained at 37 °C, 5% CO_2_ in complete medium (DMEM from Wisent; 10% Fetal Bovine Serum (Sigma, SKU: F1051); 100 U/ml penicillin G (Sigma, SKU: P3032) and 100 μg/ml streptomycin (Sigma, SKU: S9137) at a density of 10^5^ ~ 10^6^ cells/ml. For differentiation of U937 and THP-1 cells, 5.0 × 10^5^ cells/well were cultured in Corning Costar 12-well plates (Sigma, SKU: CLS3513) with 1.0 ml complete medium supplemented with 50 ng PMA (Sigma, SKU: P1585). Three days after differentiation, U937 cells were washed with PBS (Wisent, Cat. 311–425-CL) and maintained in complete medium with PMA for 2 more days. THP-1 cells were differentiated for 2 days. For differentiation of U1 cells (HIV-1 infected U937 from NIH, Cat. 165, Lot. 100218), 1 ml complete medium was supplemented with 75 ng PMA, and the cell density was increased to 7.5 × 10^5^ cells/well.

Peripheral blood mononuclear cells (PBMCs) were prepared following the protocol of Lymphoprep Density Gradient (StemCell, Cat. 07861) and as described earlier [27]. Cells were counted and cultured in Corning Costar 12-well plates at a density of 1.5 ~ 2.0 × 10^6^ cells/well in DMEM medium without supplement for 3 h. Next, the adherent monolayer was washed twice with PBS. Then, cells were maintained in complete medium supplemented with 10 ng/ml macrophage-colony stimulating factor (M-CSF, R&D, Cat. 216-MC-025) for 3 days. Cells were washed once with PBS and maintained in complete medium with the same concentration of M-CSF for 4 more days, allowing differentiation for 7 days in total before further experimentation.

### Preparation of 90K shRNA lentivirus pool and subsequent screening

Pooled screening was done as previously described [28, 29]. Briefly, 90K shRNA lentivirus pool was generated by transfecting HEK293T cells from ATCC (Cat. CRL-1573™) with psPAX2, pMD2.G, and 90K hairpin library in pLKO.1 vector with the transfection mix containing FuGENE® HD transfection reagent (Promega, Cat. E2312) and Opti-MEM (ThermoFisher, Cat. 31,985,070) for 18 h. Subsequently, the transfection media was replaced with high-Bovine Serum Albumin (BSA, Sigma, CAS. A2153-100G) growth media (DMEM containing BSA, 100 U/ml penicillin G, and 100 μg/ml streptomycin). The supernatant was harvested at 24 and 48 h. The pooled media containing lentiviruses were spun at 1000 rpm for 3 min to remove cell debris. Finally, the supernatant containing lentiviral particles were collected, aliquoted, and stored at − 80 °C. The aliquot was thawed at room temperature only once right before infection. U937 and its subclone HIV-infected, U1 cells (75 × 10^6^) were infected with 30 ml of 90K shRNA lentivirus pool. After 48 h, puromycin (Sigma, SKU: P9620) was applied to select 90K shRNA lentivirus infected cells. At 96 h after infection, live cells were collected and counted, 25 million cells were harvested as one cell pellet for a total of 2 pellets, which were designated as T_0_. The remaining cells were maintained in complete media without puromycin for 6 more days, and were harvested the same way as T_0_, and were designated as T_6_. The cells remaining after 6 days (T_6_) were differentiated with PMA for another 6 days, then trypsinized, counted and harvested as above and were designated as T_12_. The genomic DNA of cell pellets was extracted and diluted to a final concentration of 400 ng/μl for further analysis. Thus, the genome-wide screen was performed only one time in the two independent U937 and U1 cells with multiple time points, rather than as an end-point screen. Microarray probes were prepared and the shRNA drop-out from the screens were evaluated as previously described [29].

### Preparation of HIV-1 viruses from plasmid DNA

Plasmid HIV Gag-iGFP_JRFL was purchased from NIH (Cat. 12456, Lot. 130201). Plasmid pNL4.3-BAL-IRES-HSA was kindly provided by Dr. M. Tremblay from the University of Laval, Quebec [30]. Plasmid pUC-19 was purchased from ThermoFisher (Cat. SD0061) for mock infections. Plasmid DNA was transfected into chemically competent STBLE3 *E. coli* (Invitrogen, Cat. C7373–03) as per the manufacturer’s manual. Single colonies were picked directly for large volume culture in LB Medium (ThermoFisher, SKU: 12795–084) with 100 μg/ml Ampicillin (Sigma, SKU: A8351), and shaken horizontally at 30 °C for 24–30 h at 300 rpm. The bacteria were harvested, and plasmid DNA was purified with QIAGEN Plasmid Giga Kits (Cat. 12191). To produce HIV-1 and mock viruses, 50 μg plasmid DNA were transfected into 293 T cells with 125 μl of Lipofectamine™ 2000 (Invitrogen, Cat. 11668019) at a density of 15.0 × 10^6^ cells/150 mm dish (Corning, Mfr. 430599). Plasmid pUC-19 was used to produce mock viruses. Viruses in supernatant were harvested twice at 48 and 96 h, respectively. To remove cell debris, the supernatants were centrifuged at 2000 g for 15 min and filtered through 0.45 μm cellulose acetate membrane (Millipore, SKU: HAWP04700). PEG-it™ virus precipitation solution (SBI, Cat. LV825A-1) was used to precipitate viruses, and precipitants were re-suspended in 0.05 M HEPES (Sigma, SKU: H3375-25G) PBS at 1/20 volume of original supernatants, and aliquoted before storage at − 80 °C. Viruses were quantified by ELISA according to the protocol of HIV-1 p24^CA^ Antigen Capture Assay Kit from Frederick National Laboratory for Cancer Research.

### Infection of primary MDMs with HIV-eGFP and HIV-HSA viruses

All viruses in frozen stock underwent only one thaw before infection. HIV-eGFP or HIV-HSA viruses (150 ng p24) in 400 μl complete medium was applied to infect seven-day-old primary MDMs overnight. Cells were washed, and complete DMEM medium was added to make the final volume into 1.0 ml/well. For HIV-eGFP virus, cells were trypsinized and eGFP+ cells were detected by flow cytometry at day 1, 2, 3, 5, 7, and 9 post-infection. For HIV-HSA virus, cells were trypsinized, washed with PBS, blocked with 5.0 μl/10^5^cells of human FcR Blocking Reagent (Miltenyi Biotec, Order No. 130–059-901), and stained with FITC rat anti-mouse CD24 antibodies (BD Pharmingen, Mat. 561777). HSA+ cells were analyzed by flow cytometry on days 3, 5, 7, 9, 11, and 13 post-infection.

### siRNA transfection of primary MDMs and analysis of apoptosis by Annexin-V

siRNA transfection was carried out as optimized and described previously [27]. As per this optimized protocol for siRNA transfection, we employed DarmaFect 3 (Dharmacon, Cat. T-2003-03) and achieved 85% transfection efficiency with the minimum loss of cell viability in primary human MDMs [27]. Briefly, seven-day-old primary MDMs were infected with both mock and HIV-1 viruses overnight, following which cells were washed twice with PBS and maintained in complete DMEM media for 6 days. Two hours before siRNA transfection, cells were washed with PBS and maintained in 0.8 ml/well of antibiotics-free DMEM medium supplemented with 10% FBS. For siRNA transfection, 20 nmol siRNA and 1.0 μl DharmaFect 3 Transfection Reagent were added into 200 μl/well Dharmacon Transfection Medium as per the manufacturer’s manual [27]. Transfected cells were maintained for 48 ~ 72 h, trypsinized, and harvested. HIV-eGFP-infected cells were quantified by staining with Annexin-V conjugated with APC or BV711 (BD Biosciences, Cat. 550475 or 563972) followed by flow cytometry analysis. To quantify HIV-HSA-infected cells, MDMs were first blocked with 5.0 μl/10^5^ cells of human FcR blocking reagent followed by staining with FITC rat anti-mouse CD24 antibodies. Subsequently, cells were washed, counterstained with Annexin-V conjugated with BV711, and then analyzed by flow cytometry.

siRNAs of the 28 promising gene were purchased from Dharmacon (Cherry-pick Library, Cat. LP_22590 G-CUSTOM-234593). Accell non-targeting siRNA #1 (Cat. D-001910-01-05) was used as control siRNA. A mixture of *cIAP1*, *cIAP2*, and/or *XIAP* siRNA (Cat. E-004098-00-0005, E-004390-00-0005, E-004099-00-0005), or 5.0 μM of IAP antagonist AEG40730 (Tocris Bioscience, Cat. 5330) were used as positive controls. The following siRNAs were employed to silence complexes I-V: Human *Ndufa11* (126328) siRNA (Dharmacon, Cat. L-018508-01-0005) for complex I; Human *Sdha* (6389) siRNA (Dharmacon, Cat. L-009398-00-0005) for complex II; Human *Uqcrq* (27089) siRNA (Dharmacon, Cat. L-012517-01-0005) for complex III; Human *Cox7a2* (1347) siRNA (Dharmacon, Cat. L-011626-01-0005) for complex IV; Human *Atp5a1* (498) siRNA (Dharmacon, Cat. L-017064-01-0005) for complex V.

### Flow cytometry analysis for cell death by PI staining

Cell death of primary MDMs, THP-1, U937, and U1 cells was evaluated by PI staining as previously described [31]. For undifferentiated U937 and THP-1 cells, 10^5^ cells/sample were harvested and pelleted. For differentiated U937, THP-1 and primary MDMs, adherent cells were trypsinized with 0.4 ml 0.25% Trypsin-EDTA (Gibco, Cat. 25200072) for 30 min and neutralized with 0.6 ml complete medium. Cells were centrifuged at 800 g for 5.0 min, washed, re-suspended in 0.5 ml PBS with 0.5% BSA. Right before loading to flow cytometer (BD LSR FORTESSA X-20), 1.0 μl PI (Propidium Iodide, Sigma SKU: P4864) was added to the sample, vortexed and analyzed at PI channel immediately. For determination of cell death following infection with 90K shRNA lentivirus pool, differentiated or undifferentiated cells infected for 48 h were treated with puromycin for another 48 h followed by flow cytometry analysis. For the infection rate of lentivirus-eGFP, differentiated cells were infected for 24 h, washed, trypsinized and re-suspended in 0.5 ml PBS with 0.5% BSA, and analyzed by flow cytometer at GFP channel.

### SDS-PAGE and Western blot analyses

Primary MDMs were harvested and lysed in cell lysis buffer (Cell Signaling, Prod. 9803S). Proteins were quantified following Bio-Rad Protein Assay (Bio-Rad, Cat. 5000006) and analyzed in 10 ~ 15% SDS-polyacrylamide gel electrophoresis (PAGE). Precision Plus Protein Standards (BIO-RAD, Cat. 1610374) was used as the protein marker, and rabbit anti-β-actin (13E5) monoclonal antibody (Cell Signaling, Prod. 4970) was employed as a housekeeping protein. Subsequently, proteins were transferred onto PVDF membranes (Bio-Rad, Cat. 1620177), which were then blocked with 5% nonfat dried milk in PBS at 4 °C overnight. Membranes were probed with first antibody in 1% BSA in PBS for 1 h at room temperature, followed by rat anti-mouse IgG monoclonal antibody conjugated with horseradish peroxidase (Bio-Rad, Cat. MCA152) in 1% BSA. Immunoblots were visualized using Clarity Max™ Western ECL Blotting Substrates (Bio-Rad, Cat. 1705060) and imaged with Chemigenius Bio-imaging System (Syngene). Rabbit anti-CDK2 monoclonal antibody (Prod. ab32147), rabbit anti-CSTF*2*T monoclonal antibody (Prod. ab138486), rabbit anti-ZNF484 polyclonal antibody N-terminal end (Prod. ab173874), and rabbit anti-COX7A2 polyclonal antibody C-terminal end (Prod. Ab135431) were purchased from Abcam. Anti-NDUFA11 polyclonal antibody (Cat. A16239) was purchased from Abclonal Technology. Anti-SDHA polyclonal antibody (Prod. HPA 064582), and anti-ATP5A1 polyclonal antibody (Prod. HPA 044202) were purchased from Sigma Prestige Antibodies. Anti-UQCRQ polyclonal antibody (Prod. ab136679) was purchased from Abcam Technology.

### ROS production detection

Seven-day-old MDMs were infected with either mock or HIV-HSA viruses (150 ng p24) and maintained at 37 °C for 7 days. Cells were transfected with 20 nM siRNA for 48 h, harvested, and then stained with CellROX® Deep Red Reagent (Life Technologies, Cat. C10422) and FITC rat anti-mouse CD24 antibodies for 30 min at 37 °C. Cells were washed twice with 1.0 ml PBS, and then fixed with 500 μl of 1% Phosphate-Buffered Paraformaldehyde (PFA pH 7.4; FD NeuroTechnologies Inc. Cat. PF101). ROS production was analyzed by flow cytometry (BD LSRFortessa™ X-20) at APC channel and the mean fluorescent intensity (MFI) of each sample was retrieved directly from the histogram.

### Microscopy

Differentiated cells (U937, THP-1 and primary MDMs) were washed with PBS, fixed with 3.7% formaldehyde (Fisher Scientific, Cat. F79P-4), observed and imaged by fluorescence microscope (IX51 Olympus).

### Statistical analysis

Experiments on puromycin sensitivity and polybrene tolerance were repeated three times independently (*n* = 3). The apoptosis of HIV-infected primary MDMs induced by siRNA was based on 4 independent experiments from different healthy blood donors (*n* = 4). ROS production induced by HIV-1 infection and targeting respiratory complexes were based on 8 independent experiments from randomly selected 8 healthy blood donors (*n* = 8). GraphPad Prism 6.0 software was applied to calculate the mean value, standard deviation (SD), and divert the results into diagrams. P-values were calculated using student’s t-test. Plotted data represent the mean ± SD.

## Results

### Undifferentiated, PMA-differentiated U937 cells, and HIV-infected U1 cells represent effective models for genome-wide screening

To perform the unbiased genome-wide screen, we tested a series of cells including primary human MDMs, the myeloid lineage leukemic U937 cells, their subclone U1 cells (HIV-infected U937) and THP-1 cells, that are commonly used to examine macrophage-related physiological processes and can be induced to terminal monocytic differentiation by treatment with phorbol 12-myristate 13-acetate (PMA). As it is not known which of these models would be optimal for genome-wide screening, we initially optimized the parameters essential for the application of lentivirus pooled libraries. The library we employed uses puromycin as the selection marker [32], and therefore, we determined its optimal dose required for killing the highest percentages of each cell type. Puromycin effectively killed undifferentiated and PMA-differentiated U937, U1, THP-1 cells, and primary MDMs (Supp Fig. 1A-G). However, the dose required to kill PMA-differentiated U1, THP-1 and U-937 cells was 4–5 times higher than that required for undifferentiated cells. Moreover, the dose required to kill undifferentiated and PMA-differentiated U1 cells was higher than uninfected U937 cells (Supp Table 1), indicating HIV-infected cells are more resistant to puromycin-induced cell death than uninfected cells. We also tested the use of polybrene, the agent known to enhance lentivirus transduction [32], to determine its concentration that kills minimal number of the above-mentioned cells. Polybrene at concentrations of 5–10 μg/ml killed minimal numbers of U937, U1 and THP-1 cells (Supp Fig. 2A-F; Supp Table 2). However, polybrene at the lowest working concentration (5 μg/ml) was toxic to 14-day-old primary MDMs and markedly changed their morphology (Supp Fig. 2G&H; Supp Table 2), suggesting that polybrene was not suitable for MDMs. In these experiments, the 14-days -old MDMs originated from 7 days of monocytes differentiation into primary MDMs and another 7 days under HIV infection.

**Fig. 1:**
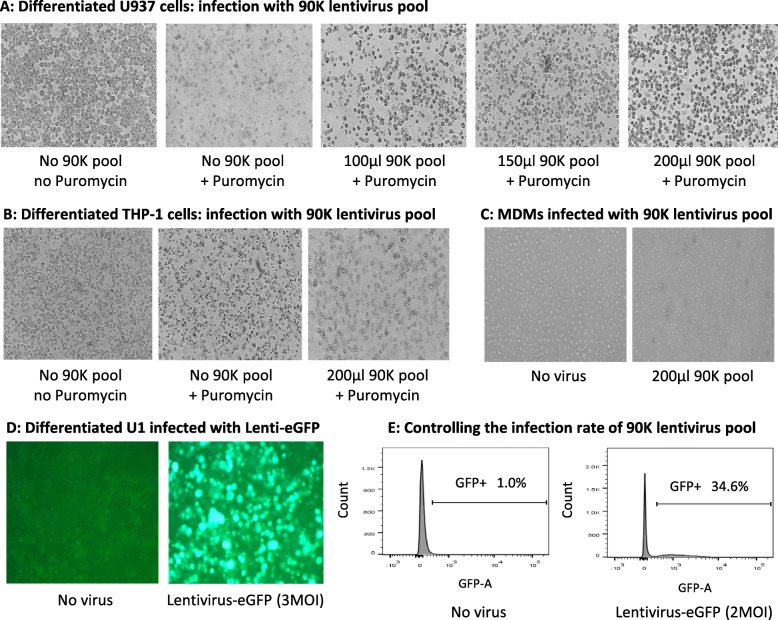
Infection of U937, THP-1, primary MDMs, and U1 cells with 90K shRNA lentivirus pool. **A** Differentiated U937 cells **B** Differentiated THP-1 cells and **C** Primary MDMs were infected with 90K shRNA lentivirus for 48 h followed by selection with puromycin for another 48 h. **D** U1 cells infected with lentivirus-eGFP were observed microscopically. **E** Controlling the infection rate of 90K shRNA Lentivirus Pool. On 90K shRNA lentivirus pool, there is no selection marker for the detection of infection rates. To control the infection rate of 90K shRNA lentivirus pool, undifferentiated U937 cells (0.5 × 10^6^) were infected with various amount of 90K shRNA lentivirus pool or 2 MOI lentivirus-eGFP for 48 h. After puromycin selection of infected cells for another 48 h, trypan blue was employed to track dead cells. When 200 μl 90K shRNA lentivirus pool and 2 MOI lentivirus-eGFP were used to infect undifferentiated U937 cells, similar numbers (approximately 35%) of live cells were counted under microscopy, indicating that the infection rate of 200 μl 90K shRNA lentivirus pool corresponded to 2 MOI

To determine if differentiated cells could be infected with 90K shRNA lentivirus pool, PMA-differentiated U937 and THP-1 cells and primary MDMs were infected with various concentrations of the pool in the presence of polybrene followed by puromycin treatment. The results show that differentiated U937 cells were effectively infected (Fig. 1A), whereas differentiated THP-1 cells could not be infected (Fig. 1B). In contrast, treatment with 90K shRNA lentivirus pool alone changed the morphology of primary MDMs leading to cell detachment and eventual cell death (Fig. 1C), suggesting that transduction with 90K shRNA lentivirus pool is not tolerated by primary MDMs.

Several HIV-1 proteins, including Nef, Vpu, and Env, down-regulate CD4 expression in macrophages to avoid super-infection [33, 34]. As a result, HIV-infected macrophages may be activated and develop resistance to secondary viral infection. Therefore, it is possible that U1 cells, a well characterized cellular model of chronically HIV-1 infected U937 cells [35], may be resistant to infection with 90K shRNA lentivirus pool. Hence, we determined if U1 cells could be infected with lentiviruses by employing lentivirus-eGFP as a reference virus. Interestingly, U1 cells were efficiently infected with lentivirus-eGFP (Fig. 1D).

On 90K shRNA lentivirus pool, there is no selection marker for the detection of infection rates. The MOI of the pool was calculated via a control test with the same infection rate as lentivirus-eGFP, of which the titer was determined by the manufacturer. To control the infection rate of 90K shRNA lentivirus pool, undifferentiated U937 cells (0.5 × 10^6^) were infected with various amount of 90K shRNA lentivirus pool or 2 MOI lentivirus-eGFP for 48 h. After puromycin selection of infected cells for another 48 h, trypan blue was employed to track dead cells. When 200 μl 90K shRNA lentivirus pool and 2 MOI lentivirus-eGFP were used to infect undifferentiated U937 cells, similar numbers (approximately 35%) of live cells were counted under microscopy, indicating that the infection rate of 200 μl 90K shRNA lentivirus pool corresponded to 2 MOI. We used 2 MOI of the lentivirus pool to infect U937 cells as only at this amount, we were able to infect 30–40% of the overall cell population (Fig. 1E) as per the protocol. Overall, the results suggest that of all the myeloid lineage cells examined, only U937 and U1 cells were suitable for the application of 90K shRNA lentiviral pool to screen for target genes, and the infection rate could be controlled to avoid double or multiple transductions of shRNAs.

### Unbiased screening identified 28 promising genes that may be essential for the survival of HIV-infected macrophages

**Fig. 2:**
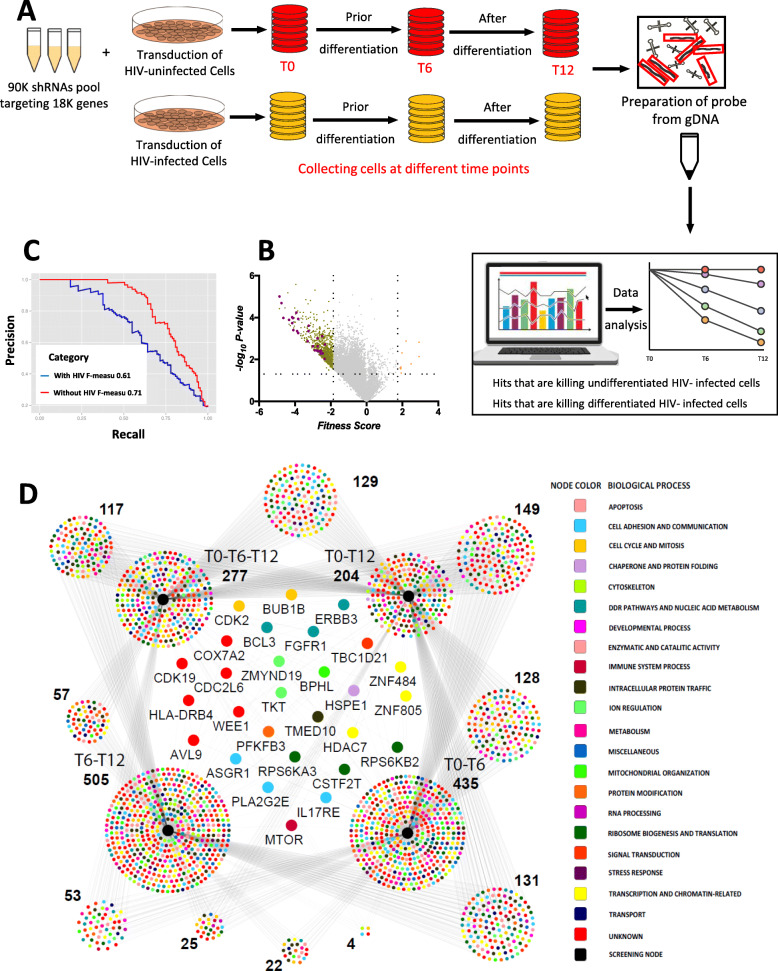
Genome-wide screening of HIV-infected and uninfected macrophages. **A** Schematic representation of the genome-wide screen. **B** Volcano plot representing the hits from the screening. The X-axis represents the fitness score and the Y-axis represents the *p*-value significance. Magenta color dots represent the final selected hits while the dark yellow represents all the significant hits from the screen. **C** Precision-Recall curve evaluating quality of the screen as described in Hart et al. 2013 (PMID: 24987113). Higher the F-measure, lower is the error rate. Usually, F > 0.5 provides a reliable screen. **D** Cytoscape representation of the hits from the screen. Since U937 and U1 cells were differentiated during the screening, the key nodes are arranged such that the hits prior to differentiation (T0-T6) and after differentiation (T6-T12) are presented in the context of overall hits. Nodes with gene names are those that were picked up in all possible conditions

We next designed our screening strategy to identify genes that, when inhibited, induce cell death selectively in HIV-infected macrophages. The workflow of the lentivirus-based pooled screen is shown in Fig. 2A. 90K shRNA lentivirus pool at MOI of 2 was applied to uninfected undifferentiated U937 and U1 cells in parallel as previously described [29]. PMA was added after the collection of T6 samples (after 6 days of culture) to allow differentiation for another 6 days (T12) (Fig. 2A). Cell pellets were harvested on indicated days (T0, T6 and T12). Thus, our experiments were designed to identify hits that kill both undifferentiated and differentiated HIV-infected cells. Genomic DNA was extracted for microarray deconvolution analysis, where gene knockdowns that caused lethality were identified by loss of shRNA-specific barcodes in microarrays [28, 29]. We used the GMAP arrays to deconvolute our pooled screen to identify genes whose loss of function caused selective lethality. The loss of specific shRNA sequences within the HIV-infected U1 cell population as compared to the U937 cell population identifies shRNA targets that selectively kill HIV-infected cells (Fig. 2B). The overall quality of the screen was measured by the published framework that depends on a reference set of essential genes as well as non-essential genes, and provides a Bayesian classifier of gene essentiality [36]. By this approach, we found our screens recorded a performance score of F-measure 0.61 and 0.71 (Fig. 2C).

Screens were analyzed considering 4 different time-points as both U937 and U1 cells differentiated after six days in culture. First, hits that caused selective lethality prior to differentiation (T0 and T6 alone) or after differentiation (taking T6 and T12 time points into consideration) were identified. We also identified hits irrespective of the differentiation status by either analyzing all time points (T0, T6 and T12) or analyzing as an end-point assay (taking T0 and T12 time points alone). We found 28 genes that were consistently identified in all possible analyses as essential for survival of HIV-infected cells. These genes are listed in Fig. 2D. This suggested that these 28 genes may represent promising targets for inducing cell death in HIV-infected macrophages. While performing the genome-wide screen in HIV-infected primary MDMs would have been ideal, the secondary infection of these cells with the lentiviral library was not successful. Given the large-scale nature of the screen in two independently controlled cell populations with two distinct conditions (differentiated and undifferentiated), we chose to perform an extensive evaluation of the 28 initially identified hits in HIV-infected primary MDMs, using the orthogonal siRNA reagents, rather than investing in additional replicates of similar large-scale screens. Therefore, we pursued an extensive evaluation of these 28 genes as a proof-of-concept approach in primary MDMs infected with HIV-eGFP [37] and HIV-HSA viruses [38] as described below.

### Establishment of HIV-infected primary MDMs using HIV-eGFP and HIV-HSA viruses for validating the top 28 hits

**Fig. 3:**
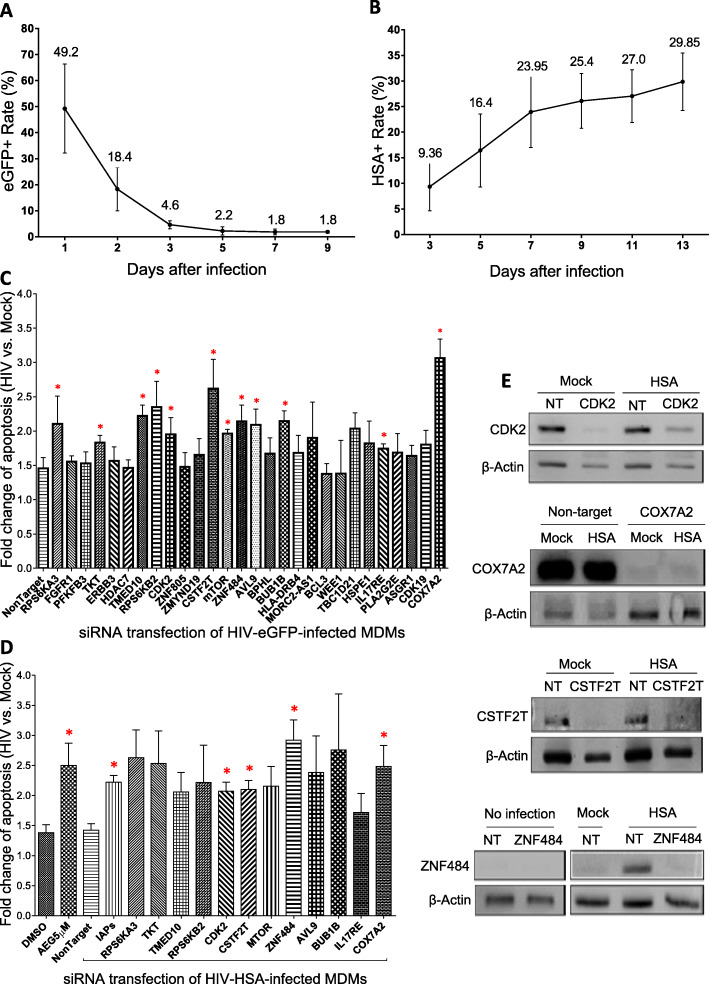
Validating promising genes with HIV-eGFP and HIV-HSA viruses by siRNAs. Kinetics of HIV-eGFP **A** and HIV-HSA **B** infection. Seven-day-old MDMs were infected with 150 ng p24 of HIV-eGFP or HIV-HSA viruses and harvested at various days after infection for flow cytometry analysis to quantify eGFP or HSA expressing cells (*N* = 3). **C** Apoptosis of HIV-eGFP-infected MDMs induced by siRNAs of 28 promising genes. Seven-day-old MDMs were infected with HIV-eGFP for 7 days and then transfected with siRNAs of 28 promising genes for 72 h. Cells were trypsinized, harvested, stained with Annexin-V antibody labelled with BV711 and apoptosis was quantified by flow cytometry. The fold change (HIV-eGFP-infected/mock-infected) of all Annexin-V+ apoptotic cells induced by each siRNA was calculated. **D** Apoptosis of HIV-HSA-infected MDMs induced by siRNAs of 12 candidate genes. Seven-day-old MDMs were infected with HIV-HSA for 7 days and then transfected with siRNAs of all 12 candidate genes for 72 h. Cells were trypsinized, harvested, stained with anti-mouse CD24 antibody (HSA-FITC) and Annexin-V-BV711 antibody and the apoptosis was analyzed with flow cytometer. Fold change (HIV-HSA-infected/mock-infected) of all Annexin-V+ apoptotic cells induced by each siRNA was calculated. The *p*-values of C and D were calculated using student’s t-test (*n* = 4). **E** siRNA transfection silenced the expression of all four identified genes in primary MDMs. Seven-day-old MDMs were infected with HIV-HSA for 7 days and then transfected with siRNAs specific for *Cdk2, Cox7a2, Cstf2t or Znf484* genes for 72 h. Cell lysates were subjected to Western blot analysis by probing with antibodies specific for CDK2, COX7A2, CSTF2T or ZNF484. The results shown are representative of three independent experiments. The full-length blots for silencing of all the four genes were shown in Supplementary Fig. 3, 4, 5, 6 and 7

The relevance of 28 genes selected from our screen was analyzed following individual transfections of matching siRNAs into primary MDMs infected with two strains of HIV-1, namely HIV-eGFP and HIV-HSA. For this, we first studied the kinetics of HIV-eGFP and HIV-HSA infection in primary MDMs. Both viruses were produced from plasmid DNA and their Env proteins were mutated to be R5-tropic to increase the infection rate in myeloid cells [39]. In addition, both viruses had the entire HIV-1 genome and expressed all individual HIV-1 proteins [30, 40]. In the HIV-eGFP viral genome, the eGFP gene is flanked by the viral Matrix and Capsid proteins, allowing for the tracking of active infection, though only capable of a single round of infection [40]. The eGFP+ cells were quantified by flow cytometry using GFP channel. In the HIV-HSA genome, an internal ribosome entry site (IRES) was constructed before the Tat gene to keep the infection active [30], and the cellular membrane-bound mouse HSA, the murine CD24 (60 amino acids), is expressed along with early viral genes upon productive infection. The presence of CD24 made it convenient to track active infections by flow cytometry using FITC labelled rat-anti-murine CD24 antibodies [38]. Similar to the results obtained by Dahabieh et al. in Jurkat T cells using single cycle HIV-eGFP construct [41], our results show that, after the infection of MDMs with 150 ng p24/well of HIV-eGFP virus, the percentage of eGFP+ cells peaked at approximately 50% within one day, but decreased quickly afterwards to 5% after 5 days of infection (Fig. 3A). In contrast, after the infection of MDMs with 150 ng p24/well of HIV-HSA virus, the percentage of infected cells varied from 5 to 10% on day 3, and increased progressively to 20–30% by day 7–10 depending upon the donor (Fig. 3B), representing an active HIV-1 infection model in macrophages.

### Assessment of genes involved in apoptosis using primary MDMs infected with HIV-eGFP and HIV-HSA viruses

Primary MDMs generated from healthy donors were infected with mock or HIV-eGFP viruses for 7 days, followed by transfection with either non-targeting siRNA or individual siRNAs specific for each of the 28 genes for 72 h. To validate the 28 promising target genes, we optimized the protocol and the reagents for siRNA transfection, and achieved 85% transfection efficiency in primary human MDMs [27]. The apoptosis of HIV-infected macrophages was analyzed by flow cytometry using Annexin-V-BV711. We performed first screen with HIV-eGFP-infected macrophages as analysis of HIV-eGFP-infected cells required single cycle of staining with Annexin-V whereas HIV-HSA-infected MDMs required double staining, first with FITC labelled CD24 antibody, and then a second staining with Annexin-V. The fold change in the abundance of apoptotic cells induced by each siRNA was calculated as the folds relative to the matching mock-infected cells (HIV-eGFP/mock-infected) (Supp. Table 3 & 4), and the results from four independent experiments were summarized (Fig. 3C). As this was the first round of analysis, we arbitrarily selected genes with *P* < 0.1 (a star * over the bar) as the candidate genes involved in the selective induction of apoptosis in HIV-eGFP-infected macrophages. Based on this cut-off, 12 genes were chosen for further analyses following infection of MDMs with HIV-HSA. MDMs infected with mock or HIV-HSA viruses for 7 days were transfected with either the non-targeting or siRNAs specific for each of the 12 selected genes for 72 h followed by flow cytometric analysis of HSA expression and apoptosis by Annexin-V staining. The second mitochondria-derived activator of caspases (Smac) mimetic (SM) AEG40730 and a mixture of siRNAs of xIAP, cIAP1, and cIAP2 were used as positive controls for induction of apoptosis [42, 43]. SMs are small peptides that competitively inhibit SM-IAPs interactions, repress anti-apoptotic functions of IAP proteins [44] and have been shown to selectively induce apoptosis of HIV-infected cells [25, 45]. The fold change of apoptotic cells induced by each siRNA was calculated as relative to the matching mock-infected controls (Fig. 3D). We selected genes with minimum variation from all the donors, and as a result provided us the *p*-values < 0.05 in contrast to genes in the set with higher variance among donors with a p-value > 0.05. The results reveal that transfection with siRNAs for four genes, namely, *Cox7a2*, *Znf484*, *Cdk2*, and *Cstf2t* exhibited statistically significant higher percentage of apoptosis in infected MDMs (*p* < 0.05), suggesting that these four genes may be targeted to selectively induce apoptosis of HIV-infected MDMs.

Finally, we verified silencing of *Cox7a2*, *Znf484*, *Cdk2*, and *Cstf2t* genes by their specific siRNAs. Primary MDMs infected with HIV-HSA or mock-infected cells for 7 days were transfected with siRNAs specific for the four identified genes for 48 h followed by Western blotting. The results show that siRNAs effectively silenced all four selected genes in HIV-HSA-infected MDMs (Fig. 3E). Interestingly, the basal expression of *Znf484* in mock-infected primary MDMs was not detectable, but its expression was upregulated after 7 days of HIV-HSA infection. *Znf484* siRNA effectively silenced its expression in HIV-HSA-infected macrophages (Fig. 3E), suggesting that the upregulation of *Znf484* was essential for the survival of HIV-infected macrophages.

### Loss of function of *Cox7a2*, *Znf484*, *Cdk2*, and *Cstf2t* genes results in specific killing of HIV-HSA-infected MDMs

The above results show that silencing of four identified genes killed significantly high numbers of total overall infected MDMs which contained both HIV-HSA-infected and uninfected HSA-negative bystander cells. However, it is imperative to determine if siRNA silencing of the identified genes selectively induced apoptosis of HIV-infected macrophages or HIV-exposed uninfected bystander macrophages or both. Even though we observed high levels of apoptosis in HIV-infected macrophages following transfections with siRNAs specific for the identified genes, it is possible that this may be, at least in part, due to the killing of uninfected bystander macrophages.

**Fig. 4:**
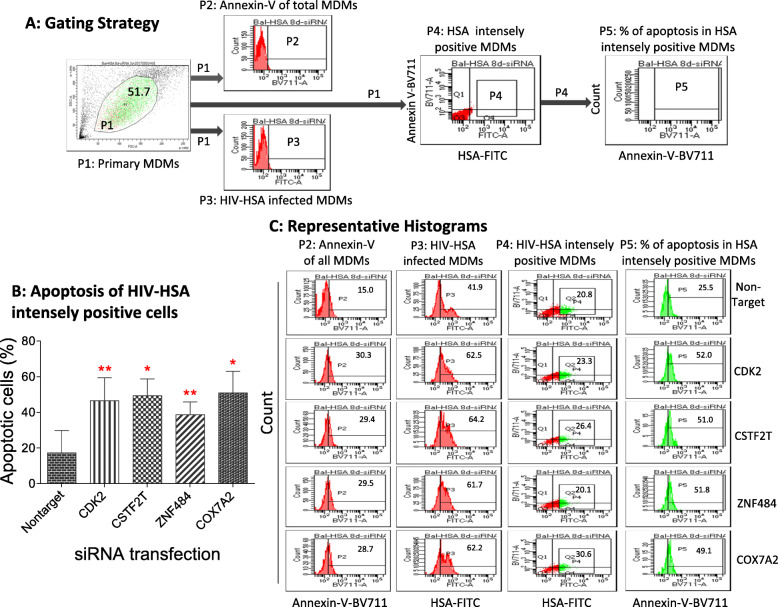
siRNAs of *Cdk2*, *Cox7a2*, *Cstf2t* and *Znf484* genes selectively induced apoptosis of intensely positive HIV-HSA-infected MDMs. Seven-day-old MDMs were infected with 150 ng p24 of HIV-HSA for 7 days and then transfected with siRNAs specific for *Cdk2, Cox7a2, Cstf2t or Znf484* genes for 72 h. Cells were trypsinized, harvested, stained with anti-mouse CD24 antibody (HSA-FITC) and Annexin-V BV711 antibody and apoptosis was quantified by flow cytometry. **A** Gating the HIV-HSA intensely positive cells. **B** Apoptosis of HSA intensely positive MDMs following transfection with siRNAs specific for the four genes. The percentage of apoptotic cells of HSA intensely positive was calculated. The p-values were calculated using student’s t-test (n = 4). **C** Representative histograms from one individual showing apoptosis of HSA intensely positive MDMs induced by siRNA transfection of the 4 identified genes

To determine if the silencing of the four identified genes killed specifically HIV-infected macrophages, we gated the population that was HSA/FITC intensively positive to rule out the non-specific weak fluorescence signals from dying HIV-exposed uninfected bystander macrophages [46, 47]. Annexin-V+ and HSA intensely positive apoptotic cells were identified as specifically killed HIV-infected macrophages. The gating strategy for selecting intensely positive HSA (mouse CD24-FITC) cells is shown in Fig. 4A. MDMs infected with mock or HIV-HSA viruses for 7 days were transfected with either non-targeting or siRNAs specific for each of the four identified genes for 72 h followed by flow cytometric analysis of apoptosis by Annexin-V staining (Fig. 4B). Quantification of Annexin-V+ and HSA+ MDMs showed that knocking down of *Cox7a2*, *Znf484*, *Cdk2*, or *Cstf2t* genes by their respective siRNAs indeed killed significantly higher percentages of total HIV-infected macrophages (30%) than non-targeting siRNA-treated (15%) macrophages (Fig. 4C left panel, P2 gate). Notably, HIV-HSA-infected MDMs transfected with control non-targeting siRNAs revealed 15% cell death. When same total MDMs (P2 gate) were stained with anti-CD24 antibody (P3 gate), we observed 40% HSA+ cells in MDMs treated with non-targeting siRNA compared to around 60% HSA+ cells in MDMs treated with siRNAs from four identified genes. Since in general 15–20% of MDMs get infected with HIV-HSA (Fig. 3A), the high percentage of HIV-HSA-positivity (40%) observed following transfection with control non-targeting siRNA and around 60% following transfection with siRNAs specific for the identified genes suggested that some HIV-HSA-negative macrophages are being killed by the siRNA transfection process. Notably, these dying cells, even though they are not HIV-infected, also emit weak FITC signals due to auto-fluorescence [48] [46, 47], and may falsely suggest the killing of higher numbers of HIV-HSA-infected MDMs (HSA+) during flow cytometry analysis. This increased HSA positivity in siRNAs treated macrophages may be attributed to the HSA signals released from the uninfected bystander cells killed by the siRNAs. The quantification of Annexin-V positive and intensely HSA-positive MDMs show that knocking down of *Cox7a2*, *Znf484*, *Cdk2*, or *Cstf2t* genes by their respective siRNAs indeed killed significantly higher percentages of intensely stained HSA+ MDMs (approximately 50%) compared to the non-targeting siRNA-treated macrophages (approximately 15–20%) (Fig. 4B). Representative histograms show the killing of intensely HIV-HSA positive MDMs (Fig. 4C).

### Knocking down *Cox7a2*, *Znf484*, *Cdk2*, and *Cstf2t* genes induces apoptosis in HIV-HSA-exposed uninfected bystander MDMs

**Fig. 5:**
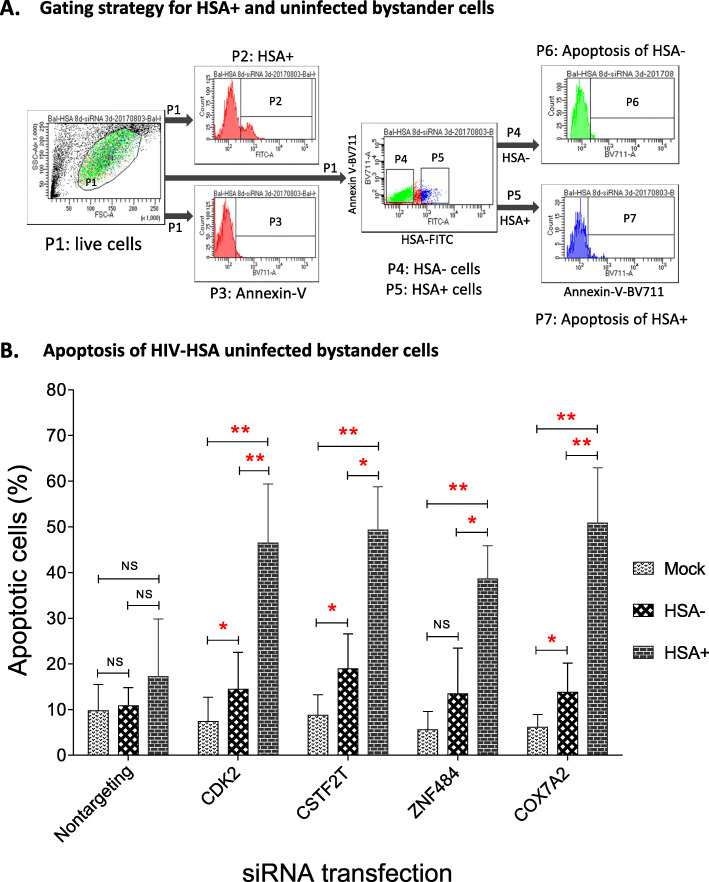
siRNAs of *Cdk2, Cox7a2, Cstf2t and Znf484* genes induced apoptosis of HIV-HSA-uninfected bystander MDMs. Seven-day-old MDMs were infected with HIV-HSA for 7 days and then transfected with siRNAs specific for *Cdk2, Cox7a2, Cstf2t or Znf484* genes for 72 h. Cells were stained with anti-mouse CD24 antibody (HSA-FITC) and Annexin-V-BV711 antibody. Specific killing of HSA+ (ie HIV-infected) and HSA- (HIV uninfected bystander) cells by siRNAs was quantified by counter staining with Annexin-V-BV711 and flow cytometry analysis. For quantification, both HSA-positive and HSA-negative cells were gated. **A** Gating strategy for apoptosis of HSA-negative bystander macrophages and HSA-positive macrophages. **B** Apoptosis of Mock-infected, HIV-HSA-exposed uninfected bystander and HIV-HSA-infected macrophages after siRNA transfection for 72 h. The *p*-values were calculated using Student’s t-test (n = 4)

The apoptosis of uninfected bystander T cells is crucial in AIDS pathogenesis [49], and is responsible for the fast and massive depletion of CD4+ T cells leading to immunodeficiency [50]. To determine if siRNA silencing of the four identified genes induced apoptosis in HIV-HSA-exposed uninfected bystander macrophages, MDMs were infected with HIV-HSA for 7 days followed by transfection with non-targeting siRNA or siRNAs specific for *Cox7a2*, *Znf484*, *Cstf2t*, and *Cdk2* for 72 h. The apoptosis was quantified by counter staining with HSA-FITC and Annexin-V-BV711. The gating strategy of HSA-positive and HSA-negative cells is shown in Fig. 5A. As expected, The *Cox7a2*, *Znf484*, *Cstf2t*, and *Cdk2* siRNAs killed significantly higher numbers of HSA+ cells compared to either the mock-infected or HIV-HSA-exposed uninfected bystander (HSA-negative) macrophages (Fig. 5B). However, killing of uninfected bystander cells was also significantly higher than the killing of the mock-infected cells with siRNAs for *Cox7a2*, *Cstf2t* and *Cdk2*, but not for *Znf484* (Fig. 5B). Overall, the results suggest that while siRNA silencing each of the four *Cox7a2*, *Cstf2t, Cdk2* and *Znf484* genes specifically killed HIV-infected macrophages, it also induced apoptotic responses in HIV-uninfected bystander macrophages.

### Silencing of respiratory complexes II and IV selectively induced apoptosis of HIV-infected macrophages through the cumulative effect of ROS production induced by HIV infection and by silencing of respiratory chain complexes II and IV

**Fig. 6:**
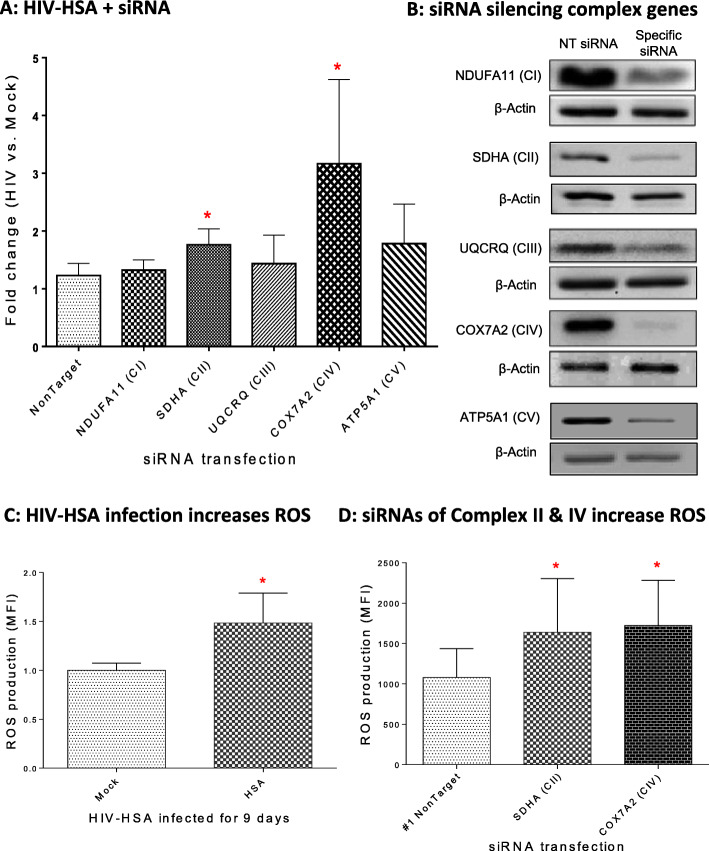
siRNAs of respiratory chain complex genes induced apoptosis of HIV-infected MDMs. **A** The apoptosis of HIV-HSA-infected MDMs after transfection with siRNA of respiratory complex I-V genes. Primary MDMs (7 days old) were infected with HIV-HSA virus followed by transfection with siRNAs of respiratory chain complex I-V genes for 72 h. The apoptosis of mock- and HIV-infected cells treated with siRNA was analyzed by flow cytometry. The fold changes of apoptosis in HIV-infected cells were calculated by normalization with mock infection (HIV-infected/mock-infected), and then compared with that of non-targeting siRNA. **B** Western blotting of siRNAs showing silencing of respiratory complex I-V genes. Seven-day-old MDMs were transfected with siRNAs specific for respiratory gene complexes I-V (CI-CV) for 72 h. Cell lysates were subjected to Western blot analysis by probing with corresponding antibodies specific for respiratory gene complexes I-V. The full-length blots for silencing the complex I-V genes are shown in Supplementary Fig. 8 ~ 12A, B, and C. **C** ROS production after HIV-HSA infection for 9 days (n = 4). **D** ROS production induced by siRNA silencing of *Sdha* (complex II) and *Cox7a2* (complex IV) (*n* = 8). Seven-day-old primary MDMs were treated with siRNA for 2 days. MDMs were harvested and stained with CellROX red followed by flow cytometry analysis at APC channel. The *p*-values were calculated using student’s t-test (n = 8)

Mitochondria are well known to play a central role in cell survival [51–53], and COX7A2 is the terminal component of mitochondrial respiratory complex IV (cytochrome c oxidase) [54]. The respiratory chain in the mitochondria is comprised of 5 complexes, namely complexes I-V. Thus, we determined if silencing the respiratory chain complexes I-V could also induce apoptosis in HIV-infected macrophages. The siRNAs of *Ndufa11* [55], *Sdha* [55], *Uqcrq* [56], and *Atp5a1* [57] have been shown to effectively block the functions of complexes I, II, III, IV, and V, respectively. Our observations revealed that similar to silencing *Cox7a2,* siRNA silencing the *Sdha* subunit of complex II induced significantly greater apoptotic responses in HIV-HSA-infected primary MDMs compared to mock-infected cells (Fig. 6A). Western blot analysis showed that siRNAs specific for complexes I-V effectively silenced all five selected genes in primary MDMs (Fig. 6B).

**Fig. 7:**
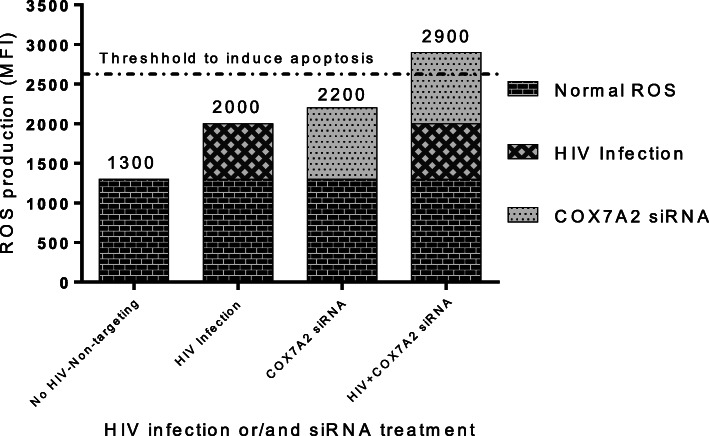
Model depicting targeting *Cox7a2* induced apoptosis of HIV-infected MDMs. Without HIV infection and without *Cox7a2* knockdown, ROS induced by cells themselves cannot induce apoptosis. HIV infection alone and targeting *Cox7a2* alone increased ROS production but cannot cause apoptosis. Only when ROS production reaches the threshold (dash line), do the cells undergo apoptosis. Silencing of *Cox7a2* in HIV-infected macrophages results in ROS production that surpasses the threshold required to induce apoptosis

Subsequently, we investigated the mechanism of targeting *Cox7a2 *induced apoptosis of HIV-infected macrophages. Silencing *Cox7a2* enhances ROS production by interfering with the respiratory chain complex [58]. Likewise, HIV infection also increases ROS in both in vitro and in vivo infections and in monocytes of HIV-infected individuals [59, 60]. Moreover, the levels of superoxide dismutase (SOD), an enzyme known to break down potentially harmful oxygen molecules in cytoplasm, are reduced in monocytes of HIV-infected patients [61]. The impaired functioning of the mitochondrial respiratory chain complexes is also known to increase ROS production [62–65]. Oxidative stress has also been shown to induce apoptosis in various cell types [64, 66]. Therefore, we next determined whether HIV-HSA infection and interfering with respiratory complexes II and IV enhanced ROS production in macrophages. Primary human MDMs were infected with HIV-HSA for 7 days, transfected with siRNA for *Sdha* (complex II) and *Cox7a2* (complex IV) for 48 h, and then stained with CellROX® and analyzed by flow cytometry. Both HIV-HSA infection and siRNA silencing of *Sdha* (complex II) and *Cox7a2* (complex IV) significantly enhanced ROS production in primary MDMs (Fig. 6C & D). However, total ROS production in primary MDMs caused by siRNA silencing of *Sdha* or *Cox7a2* and HIV infection was undetectable, since targeting *Sdha* or *Cox7a2* in HIV-infected macrophages induced apoptosis of HIV-infected macrophages which turned into cellular debris. Since either HIV infection alone or targeting *Cox7a2* alone in uninfected macrophages did not induce cell death of macrophages, ROS stress caused by HIV infection alone or by targeting *Cox7a2* alone (Fig. 6C, D) is not sufficient to trigger apoptosis. We believe that the accumulation of ROS production induced by silencing *Cox7a2* and HIV infection surpassed the ROS threshold required to induce apoptosis in HIV-infected macrophages (Fig. 7).

## Discussion

In this study, we applied a pooled shRNA-based genome-wide screen to identify genes that can be targeted to selectively induce apoptosis in HIV-infected macrophages. We found that silencing *Cox7a2*, *Znf484*, *Cdk2*, and *Cstf2t* genes could specifically kill HIV-infected macrophages. Furthermore, an attempt to elucidate the mechanism governing *Cox7a2-*mediated apoptosis of HIV-infected macrophages revealed that targeting respiratory chain complex II and complex IV genes (Sdha or *Cox7a2*) also selectively induced apoptosis of HIV-infected macrophages, and the mechanism is most likely through enhanced ROS production.

There are at least 19,000 genes in a complete haploid set of human genome [67], which make the screening process for target genes a big challenge. To address this challenge, we employed an unbiased, pooled shRNA-based screening strategy based on 90K shRNA lentivirus pooled library, to screen genes that could be targeted to selectively induce apoptosis in HIV-infected macrophages. This technology was originally designed and successfully used to identify target genes for anti-cancer therapy [28, 29]. Herein, we demonstrated that this technology can be customized to screen novel gene targets for selective killing of HIV-infected macrophages. Since macrophages are not the only HIV cellular reservoir [68], this technology may also be applied to other HIV cellular reservoirs such as memory T cells, and also for screening for novel gene targets for other human or animal diseases.

Since the 90K lentivirus pool has been proved to be toxic to primary MDMs, we performed our initial screening for genes responsible for the survival of HIV-infected macrophages in undifferentiated and PMA-differentiated U937 and U1 cells. We determined 28 promising genes that appeared to be crucial for the survival of U1 cells. The screening of target genes in U937 and U1 cells was not optimal, as these cells are of leukemic origin, and different molecular mechanisms might be governing apoptosis in HIV-infected primary macrophages from leukemic cells. Therefore, by screening for target genes in the U937/U1 model and validating apoptotic effects directly in primary MDMs, we bypassed technical barriers, and collected biologically relevant and reliable data. Moreover, we employed two distinct HIV strains namely, HIV-eGFP and HIV-HSA [40, 69–71], to infect primary macrophages. Both HIV strains expressed the complete HIV-1 genome and distinct selection markers to track HIV-infected macrophages for cell death analysis [38, 72]. We employed HIV-eGFP model for initial screening of promising genes which were later validated by HIV-HSA-infected model. HIV-HSA-infected 5 to 25% macrophages in contrast to 2–5% macrophages infected with HIV-eGFP with single round of infectivity over the period of 10 days post-infection. Thus, these two infection models provided an opportunity to study HIV-specific killing of HIV-infected macrophages.

The optimal transfection of primary MDMs with siRNAs involved a variety of technical issues, such as individual variations [73], restriction factors [74] and low rate of HIV infection in primary MDMs [16, 75–77]. In addition, primary MDMs are, in general, difficult to transfect with siRNAs. We rigorously optimized this technique by selecting the most suitable transfection reagents from Dharmacon with the transfection efficiency of 85% and with the minimum loss of cell viability [27]. Consistent results could be observed only if the infection rate of 15–25% was achieved.

Our observations reveal that silencing *Cox7a2*, *Znf484*, *Cdk2*, and *Cstf2t* genes induced cell death preferentially in HIV-infected primary macrophages. All four identified genes play important roles in cancer development and may be targeted for cancer therapy. For example, siRNA silencing of *Cdk2* and *Csf2t* genes induced apoptosis of neuroblastoma cells and lung cancer cells, respectively [78, 79]. *Znf484* has been identified as a cancer-associated gene and is involved in hepatic tumorigenesis [79]. Likewise, *Cox7a2* expression has been used as a marker for monitoring cancer development, predicting regional lymph node metastasis, and disease outcome [80]. The information indicates that each of the 4 identified genes plays a crucial role in the cell survival.

We have shown that siRNA silencing each of the four identified genes induced specific killing of HIV-infected macrophages as detected and quantified by the killing of HSA intensely positive cells. Moreover, targeting these single genes also killed significantly higher number of HIV-HSA-infected MDMs than HIV-HSA-exposed uninfected bystander macrophages, further indicating the specific killing of the HIV-infected macrophages. Keeping in view that the apoptosis of uninfected bystander T cells plays a crucial role in HIV pathogenesis [49, 50], silencing the four identified genes also induced significant apoptosis of uninfected bystander macrophages in the active HIV-HSA infection model. Although the mechanism for killing of uninfected bystander macrophages is not clear, it is possible that HIV proteins such as Vpr and Env secreted into the supernatants may prime bystander MDMs for apoptotic death [81]. The mechanism of the apoptosis of uninfected bystander macrophages needs further investigation.

We have observed that silencing of identified genes by their respective siRNAs killed significantly higher 30–40% of total HIV-infected macrophages compared to 15% killed by the non-targeting siRNA. Furthermore, staining of siRNA transfected cells by anti-CD24 antibody revealed 40% HSA+ cells in MDMs treated with non-targeting siRNA as opposed to 60% HSA+ cells in MDMs treated with siRNA from four identified genes. Since in general only 15–20% of MDMs got infected with HIV-HSA, the high percentage of HIV-infected macrophages killed by the siRNAs suggest that some HIV-HSA negative macrophages are being killed by the siRNAs. This increased HSA positivity in siRNAs transfected macrophages maybe attributed to the auto fluorescent signals released from the dying cells killed by the siRNAs for the following reasons: 1. siRNA transfection is known to non-specifically kill cells; 2. siRNAs are not known to be HIV-activating agents or induce HIV transcription; 3. silencing by siRNAs specific for the identified genes did cause killing of HIV-HSA-exposed uninfected bystander cells and finally 4. dying cells are known to emit FITC signals [46, 47].

The identification of four biologically unrelated genes, *Cox7a2*, *Znf484*, *Cdk2*, and *Cstf2t* as targets and for inducing the selective cell death suggests that the related cell death responses may be mediated through distinct apoptotic mechanisms. The apoptosis of HIV-infected macrophages induced by silencing *Cdk2, Cstf2t*, and *Znf484* genes may be mediated through cell cycle regulation [82], internal oligoadenylation and RNA recognition [83], and gene transcription and translation [84], respectively. Further studies are needed to investigate in detail the mechanisms governing apoptosis induced by the suppression of these genes in HIV-infected macrophages. In regard to this, it is interesting that *Znf484* gene represents a class of target genes, whose upregulation following HIV infection was found to be essential for the survival of infected cells.

COX7A2 constitutes the terminal component of mitochondrial respiratory chain complex IV (cytochrome c oxidase) [54]. The mitochondria carry out cellular respiration through the respiratory chain located on the inner mitochondrial membrane (IMM). It is comprised of 5 complexes, namely complexes I-V. The malfunction of the respiratory complexes has been shown to increase ROS production [62–65], and ROS stress induces apoptosis in various cell types [64, 66]. Silencing *Cox7a2*, essentially complex IV, induced apoptosis selectively in HIV-infected macrophages, which is likely attributed to the cumulative effect of ROS produced following HIV infection and following *Cox7a2* silencing. Our results confirmed reports that HIV infection of macrophages and silencing of *Cox7a2* enhanced ROS production perhaps by interfering with the respiratory chain [58–60]. The observations that either HIV infection itself or silencing of *Cox7a2* in uninfected macrophages did not trigger cell death, whereas silencing *Cox7a2* in HIV-infected macrophages induced cell death, suggesting that ROS stress caused by either HIV infection or by *Cox7a2* silencing is not sufficient to trigger apoptosis. It is reasonable to speculate that the accumulated ROS produced by silencing *Cox7a2* and HIV infection surpassed the threshold needed to selectively induce apoptosis of HIV-infected MDMs (Fig. 7).

Subsequent to the identification of the COX7A2 subunit, a component of respiratory chain complex IV, involved in apoptosis of HIV-infected macrophages, our results show that silencing of *Sdha* of complex II also induced apoptosis in HIV-infected macrophages, possibly through HIV interaction with the respiratory chain and ROS production. Although complexes I and II are parallel electron transport pathways, unlike complex I, complex II is not a part of the respiratory super-complexes, does not transport protons to the intermembrane space, and contributes less energy to the overall electron transport process [85]. The precise roles of the respiratory complexes II and IV and ROS production in apoptosis of HIV-infected macrophages needs further investigation.

We also searched for chemical compounds specific for targeting proteins encoded by the four identified genes to kill HIV-infected macrophages. Unfortunately, all the commercially available chemical inhibitors of these proteins were found to be non-specific and highly toxic. For example, sodium cyanide and sodium azide, the compounds best known for inhibiting electron transfer in complex IV [86] are broad spectrum and highly poisonous biocides [87]. Currently, chemical compounds which specifically inhibit the proteins of interest are not commercially available. Therefore, designing, developing, and testing effective drugs/chemical inhibitors for the identified gene products which efficiently penetrate macrophage membranes needs further investigation with therapeutic goals in perspective.

In summary, the outcomes of this investigation highlight the potential of the identified genes, including that of the respiratory chain complexes II and IV as targets for eliminating HIV-infected macrophages in physiological environment. The development of specific inhibitors of the corresponding protein components might be translated into clinical interventions aimed at eradicating HIV-infected macrophages.

## Supplementary Information

**Additional File 1.**

**Additional File 2.** (PPTX 2407 kb)

## Data Availability

The datasets generated and/or analyzed in this study are available in the FlowRepository, http://flowrepository.org/id/RvFrErT1D1daWwECLXyWHgqYNlhtEdladtNRZimA8COZNoFJVEZ7rN4uJYq86yYo. All the references included in this article are publicly published papers and all the reagents used, including 90K lentiviral shRNA pool, are commercially available.

## Abbreviations

AIDS: Acquired Immunodeficiency Syndrome
ART: Anti-retroviral Therapy
ATCC: American Type Culture Collection
BSA: Bovine Serum Albumin
CD: Cluster of Differentiation
cIAP: cellular Inhibitor of Apoptosis Protein
DMEM: Dulbecco’s Modified Eagle Medium
EDTA: Ethylenediaminetetraacetic Acid
eGFP: enhanced Green Fluorescent Protein
ELISA: Enzyme-linked Immunosorbent Assay
PBS: Phosphate Buffered Saline
HIV: Human Immunodeficiency Virus
HIV-eGFP: HIV viruses made from plasmid HIV Gag-iGFP_JRFL
MDMs: Monocyte-derived Macrophages
HIV-HSA: HIV viruses made from plasmid pNL4.3-BAL-IRES-HSA
HSA: Heat Stable Antigen (mouse CD24)
IMM: Inner Mitochondrial Membrane
M-CSF: Macrophage-colony Stimulating Factor
MFI: Mean Fluorescent Intensity
MOI: Multiplicity of Infection
NIH: National Institutes of Health
PAGE: Polyacrylamide Gel Electrophoresis
PBMCs: Peripheral Blood Mononuclear Cells
PMA: Phorbol 12-myristate 13-acetate
ROS: Reactive Oxygen Species
SD: Standard Deviation
SDS: Sodium Dodecyl Sulfate
siRNAs: short interfering RNAs
shRNA: short hairpin RNAs
SM: SMAC Mimetics
SOD: Superoxide Dismutase
TNF: Tumor Necrosis Factor
TRAIL: TNF Related Apoptosis Inducing Ligand
XIAP: X-linked Inhibitor of Apoptosis Protein
