# Supplementary material for: Identification of novel genes involved in apoptosis of HIV-infected macrophages using unbiased genome-wide screening
Source: BMC Infect Dis. 2021 Jul 7;21:655. doi: 10.1186/s12879-021-06346-7 (PMC8261936; doi:10.1186/s12879-021-06346-7)
Supplement: Supplementary file 1 — Additional File 1. [file 12879_2021_6346_MOESM1_ESM.docx]

**Supplementary Tables**

| Cells | **Calculating days for Puromycin selection** | | | **Results** |
| --- | --- | --- | --- | --- |
|  | Days of differentiation | Left untreated  (Reserved for 90K shRNA) | Puromycin  treatment | Puromycin concentration to  reach the plateau of cell death |
| U937 undifferentiated | N/A | 2 days | 2 days | 1.5 µg/ml |
| U937 PMA- differentiated | 5 days | 2 days | 2 days | 5.0 µg/ml |
| U1 undifferentiated | N/A | 2 days | 2 days | 2.5 µg/ml |
| U1 PMA-differentiated | 5 days | 2 days | 2 days | 9.5 µg/ml |
| THP-1 undifferentiated | N/A | 2 days | 2 days | 2.5 µg/ml |
| THP-1 PMA- differentiated | 2 days | 2 days | 2 days | 9.5 µg/ml |
| Primary MDMs (M-CSF 7 days, HIV infection 7 days) | 14 days | 2 days | 2 days | 19.0 µg/ml |

**Supplementary Table 1: Puromycin sensitivity of myeloid lineage cells.** As per the protocol of 90K shRNA lentivirus pool technology, cells were treated with 90K shRNA lentivirus pool for two days. To determine puromycin sensitivity, various differentiated and undifferentiated myeloid cells were left untreated for 2 days followed by treatment with puromycin at various concentrations to determine the lowest puromycin concentration required for attaining the plateau of cell death. All results in supplemental Figure 1 are summarized in the last column of this table.

|  | **Cells** | **Polybrene working concentration recommended** |  |
| --- | --- | --- | --- |
|  | **U937 undifferentiated** | 5-8 µg/ml |  |
|  | **U937 differentiated 5 days** | 5-10 µg/ml |  |
|  | **U1 undifferentiated** | 5-10 µg/ml |  |
|  | **U1 differentiated 5 days** | 5-10 µg/ml |  |
|  | **THP-1 undifferentiated** | 5-10 µg/ml |  |
|  | **THP-1 differentiated 2 days** | 5-10 µg/ml |  |
|  | **Primary MDMs** | N/A |  |
|  |  |  |  |

**Supplementary Table 2: Summary of Polybrene tolerance of undifferentiated and PMA-differentiated U937, U1, THP-1 and primary MDMs.** The undifferentiated and PMA-differentiated U937, U1, THP-1 cells and primary MDMs were treated with various concentrations of polybrene to determine the highest concentration that causes minimal levels of cell death. The results shown in supplementary Figure 2 are summarized into this table.

| **A: Raw data of the apoptosis induced by siRNAs (Gene 1-14)** | | | | | | | | | | | | | | | | | | | |
| --- | --- | --- | --- | --- | --- | --- | --- | --- | --- | --- | --- | --- | --- | --- | --- | --- | --- | --- | --- |
| **siRNA** | | **#1NT** | | **RPS6KA3** | **FGFR1** | **PFKFB3** | **TKT** | **ERBB3** | **HDAC7** | **TMED10** | **RPS6KB2** | **CDK2** | **ZNF805** | | | **ZMYND19** | **CSTF2T** | **mTOR** | **ZNF484** |
| **Donor-1** | **Mock** | 5.6 | | 4.1 | 4.2 | 9.1 | 10.2 | 7.5 | 4.4 | 2.8 | 2.3 | 10.8 | 8.8 | | | 8.1 | 2.6 | 7.9 | 11.5 |
|  | **HIV-eGFP** | 8.3 | | 10.6 | 5.9 | 12.9 | 16.9 | 13.3 | 7.1 | 5.8 | 7.7 | 19.4 | 12.2 | | | 10.1 | 8.3 | 15.4 | 19.7 |
| **Donor-2** | **Mock** | 4.6 | | 3.6 | 4.3 | 4.7 | 5.6 | 4.06 | 4.6 | 3.6 | 3.3 | 3.5 | 5.9 | | | 5.7 | 5.4 | 4.2 | 4.7 |
|  | **HIV-eGFP** | 7.8 | | 7.7 | 6.7 | 6.5 | 11.3 | 4.05 | 6.8 | 8.3 | 6.1 | 8.4 | 5.7 | | | 10.6 | 14 | 8.4 | 11.3 |
| **Donor-3** | **Mock** | 6.4 | | 5 | 5.8 | 5.4 | 5.6 | 6.1 | 6.1 | 4.9 | 5.2 | 5.7 | 5.2 | | | 5.7 | 7.5 | 6 | 6.6 |
|  | **HIV-eGFP** | 10.5 | | 13.7 | 10.4 | 10.9 | 11.2 | 9.6 | 10 | 12.8 | 12.9 | 13 | 10.2 | | | 12.7 | 24.5 | 10.8 | 17.6 |
| **Donor-4** | **Mock** | 10.5 | | 15.2 | 8.9 | 10 | 9.1 | 6.8 | 9.2 | 11.3 | 7.6 | 8.8 | 8.6 | | | 8.3 | 9 | 8.2 | 12.3 |
|  | **HIV-eGFP** | 10.5 | | 14.4 | 13.1 | 13 | 15 | 13.1 | 10.4 | 21.5 | 13.1 | 11.7 | 13.8 | | | 10.5 | 12.9 | 17.2 | 22 |
| **B: Fold change calculation** | | | | | | | | | | | | | | | | | | | |
| **siRNA** | | **#1NT** | | **RPS6KA3** | **FGFR1** | **PFKFB3** | **TKT** | **ERBB3** | **HDAC7** | **TMED10** | **RPS6KB2** | **CDK2** | **ZNF805** | | | **ZMYND19** | **CSTF2T** | **mTOR** | **ZNF484** |
| **Donor-1** | | 1.48 | | 2.59 | 1.40 | 1.42 | 1.66 | 1.77 | 1.61 | 2.07 | 3.35 | 1.80 | 1.39 | | | 1.25 | 3.19 | 1.95 | 1.71 |
| **Donor-2** | | 1.70 | | 2.14 | 1.56 | 1.38 | 2.02 | 1.00 | 1.48 | 2.31 | 1.85 | 2.40 | 0.97 | | | 1.86 | 2.59 | 2.00 | 2.40 |
| **Donor-3** | | 1.64 | | 2.74 | 1.79 | 2.02 | 2.00 | 1.57 | 1.64 | 2.61 | 2.48 | 2.28 | 1.96 | | | 2.23 | 3.27 | 1.80 | 2.67 |
| **Donor-4** | | 1.00 | | 0.95 | 1.47 | 1.30 | 1.65 | 1.93 | 1.13 | 1.90 | 1.72 | 1.33 | 1.60 | | | 1.27 | 1.43 | 2.10 | 1.79 |
| **C: Mean value and p-value calculation** | | | | | | | | | | | | | | | | | | | |
| **Mean Value** | | 1.45 | 2.10 | | 1.56 | 1.53 | 1.83 | 1.57 | 1.47 | 2.22 | 2.35 | 1.95 | | 1.48 | 1.65 | | 2.62 | 1.96 | 2.14 |
| **P Value** | |  | 0.104 | | 0.512 | 0.673 | 0.032 | 0.761 | 0.904 | 0.004 | 0.087 | 0.017 | 0.937 | | | 0.333 | 0.031 | 0.091 | 0.026 |

**Supplementary Table-3:** **HIV-eGFP data analysis of apoptosis induced by siRNAs of 28 promising genes (Gene 1-14).** A: Raw data of the apoptosis induced by siRNAs. All the digits are the percentage (%) of apoptosis of MDMs induced by 20nm siRNA. B: Fold change calculation by the formula Fold Change = percentage (%) of apoptosis of HIV-eGFP-infected samples/percentage (%) of apoptosis of Mock-infected sample. C: Mean value and p-value calculation following the formulas of Microsoft Excel. Genes with p-value ≤ 0.1 were identified as promising genes.

| **A: Raw data of the apoptosis induced by siRNAs (Gene 15-28)** | | | | | | | | | | | | | | | | |
| --- | --- | --- | --- | --- | --- | --- | --- | --- | --- | --- | --- | --- | --- | --- | --- | --- |
| **siRNA** | | **#1NT** | **AVL9** | **BPHL** | **BUB1B** | **HLA-DRB4** | **MORC2-AS1** | **BCL3** | **WEE1** | **TBC1D21** | **HSPE1** | **IL17RE** | **PLA2G2E** | **ASGR1** | **CDK19** | **COX7A2** |
| **Donor-1** | **Mock** | 5.6 | 3.6 | 3.5 | 9.5 | 3.5 | 2.5 | 10 | 12.4 | 2.8 | 3.6 | 4.3 | 11.6 | 10.3 | 3.8 | 4.6 |
|  | **HIV-eGFP** | 8.3 | 8.3 | 4.9 | 20.5 | 7.7 | 8.4 | 12.2 | 11.1 | 7.1 | 9.7 | 8.1 | 14.2 | 18.3 | 6.7 | 17.2 |
| **Donor-2** |  | 4.6 | 4.1 | 5.1 | 4.4 | 5.8 | 7.9 | 4.5 | 5.5 | 4.9 | 4 | 4.2 | 5.7 | 4.2 | 3 | 5.7 |
|  |  | 7.8 | 9 | 5.9 | 9.6 | 6.2 | 7.9 | 4.8 | 4.2 | 7.1 | 5.7 | 7.4 | 11.2 | 5.1 | 6.2 | 13.7 |
| **Donor-3** | **Mock** | 6.4 | 4.8 | 5.6 | 4.8 | 4.2 | 5.1 | 5.1 | 3.5 | 5.6 | 4.1 | 4.1 | 4.4 | 6.3 | 3.9 | 5.4 |
|  | **HIV-eGFP** | 10.5 | 11.7 | 11.5 | 8.4 | 8.3 | 9.8 | 8.9 | 9.9 | 12.5 | 7.9 | 7.3 | 10.3 | 10.3 | 8.4 | 15.7 |
| **Donor-4** | **Mock** | 10.5 | 8.8 | 6 | 6.8 | 6.9 | 8.1 | 7.4 | 9.7 | 6.4 | 9.2 | 7.4 | 9.7 | 8.1 | 8.3 | 9.4 |
|  | **HIV-eGFP** | 10.5 | 12.4 | 12.4 | 16.9 | 10.2 | 10.8 | 10.9 | 10 | 12.3 | 11.3 | 11.5 | 11.8 | 15.6 | 10.2 | 30.1 |
| **B: Fold change calculation** | | | | | | | | | | | | | | | | |
| **siRNA** | | **#1NT** | **AVL9** | **BPHL** | **BUB1B** | **HLA-DRB4** | **MORC2-AS1** | **BCL3** | **WEE1** | **TBC1D21** | **HSPE1** | **IL17RE** | **PLA2G2E** | **ASGR1** | **CDK19** | **Cox7A2** |
| **Donor-1** | | 1.48 | 2.31 | 1.40 | 2.16 | 2.20 | 3.36 | 1.22 | 0.90 | 2.54 | 2.69 | 1.88 | 1.22 | 1.78 | 1.76 | 3.74 |
| **Donor-2** | | 1.70 | 2.20 | 1.16 | 2.18 | 1.07 | 1.00 | 1.07 | 0.76 | 1.45 | 1.43 | 1.76 | 1.96 | 1.21 | 2.07 | 2.40 |
| **Donor-3** | | 1.64 | 2.44 | 2.05 | 1.75 | 1.98 | 1.92 | 1.75 | 2.83 | 2.23 | 1.93 | 1.78 | 2.34 | 1.63 | 2.15 | 2.91 |
| **Donor-4** | | 1.00 | 1.41 | 2.07 | 2.49 | 1.48 | 1.33 | 1.47 | 1.03 | 1.92 | 1.23 | 1.55 | 1.22 | 1.93 | 1.23 | 3.20 |
| **C: Mean value and p-value calculation** | | | | | | | | | | | | | | | | |
| **Mean Value** | | 1.45 | 2.09 | 1.67 | 2.14 | 1.68 | 1.90 | 1.38 | 1.38 | 2.03 | 1.82 | 1.75 | 1.69 | 1.64 | 1.80 | 3.06 |
| **P Value** | |  | 0.009 | 0.577 | 0.098 | 0.499 | 0.460 | 0.763 | 0.882 | 0.141 | 0.329 | 0.083 | 0.322 | 0.578 | 0.011 | 0.024 |

**Supplementary Table-4: HIV-eGFP data analysis of apoptosis induced by siRNAs of 28 promising genes (Gene 15-28).** A: Raw data of the apoptosis induced by siRNAs. All the digits are the percentage (%) of apoptosis of MDMs induced by 20nm siRNA. B: Fold change calculation by the formula Fold Change = The percentage (%) of apoptosis of HIV-eGFP-infected samples/the percentage (%) of apoptosis of Mock-infected sample. C: Mean value and p-value calculation following the formulas of Microsoft Excel. Genes with p-value ≤ 0.1 were identified as promising genes.
