# Supplementary material for: Identification of novel genes involved in apoptosis of HIV-infected macrophages using unbiased genome-wide screening
Source: BMC Infect Dis. 2021 Jul 7;21:655. doi: 10.1186/s12879-021-06346-7 (PMC8261936; doi:10.1186/s12879-021-06346-7)
Supplement: Supplementary file 2 — Additional File 2. (PPTX 2407 kb) [file 12879_2021_6346_MOESM2_ESM.pptx]

## Slide 1
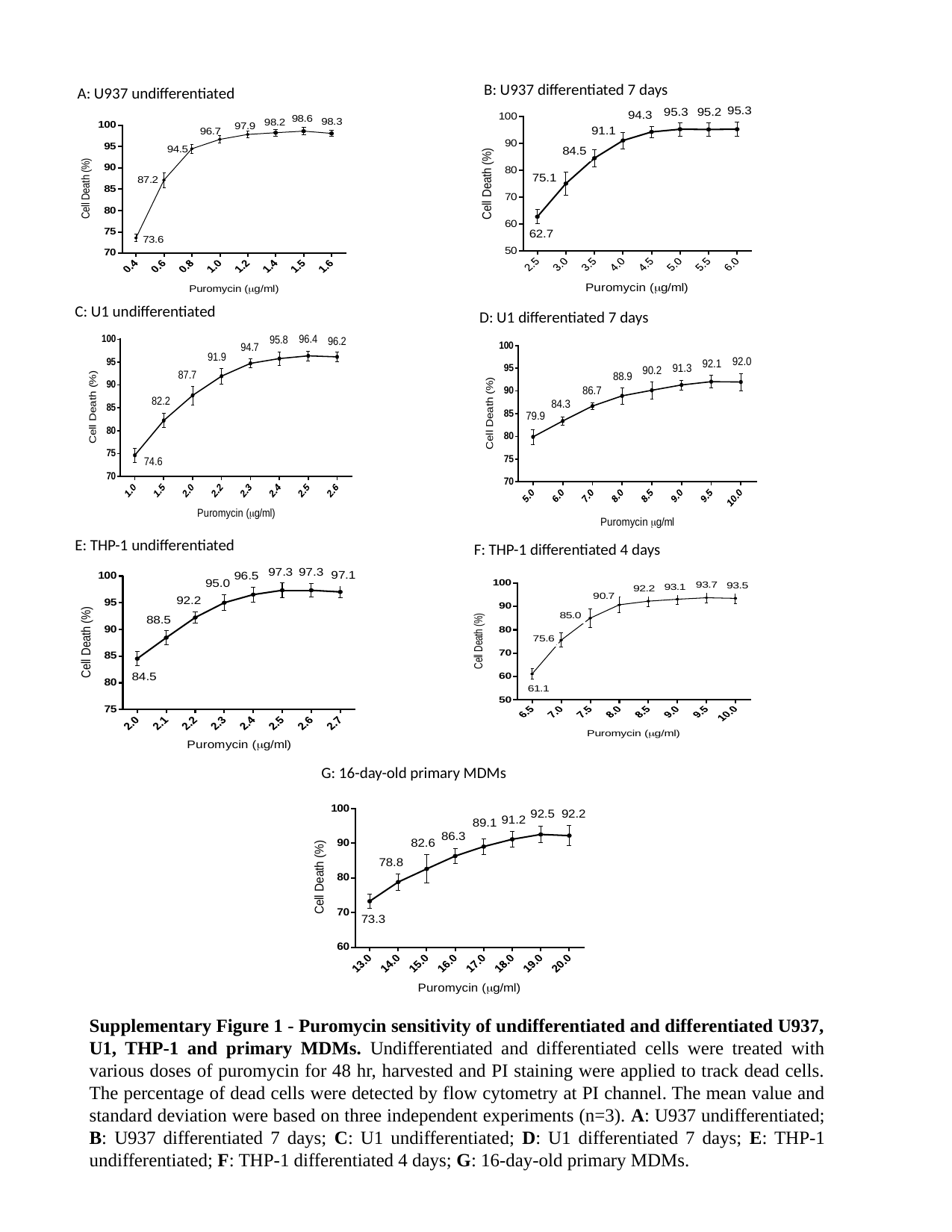

B: U937 differentiated 7 days
A: U937 undifferentiated
C: U1 undifferentiated
D: U1 differentiated 7 days
E: THP-1 undifferentiated
F: THP-1 differentiated 4 days
G: 16-day-old primary MDMs
Supplementary Figure 1 - Puromycin sensitivity of undifferentiated and differentiated U937, U1, THP-1 and primary MDMs. Undifferentiated and differentiated cells were treated with various doses of puromycin for 48 hr, harvested and PI staining were applied to track dead cells. The percentage of dead cells were detected by flow cytometry at PI channel. The mean value and standard deviation were based on three independent experiments (n=3). A: U937 undifferentiated; B: U937 differentiated 7 days; C: U1 undifferentiated; D: U1 differentiated 7 days; E: THP-1 undifferentiated; F: THP-1 differentiated 4 days; G: 16-day-old primary MDMs.

## Slide 2
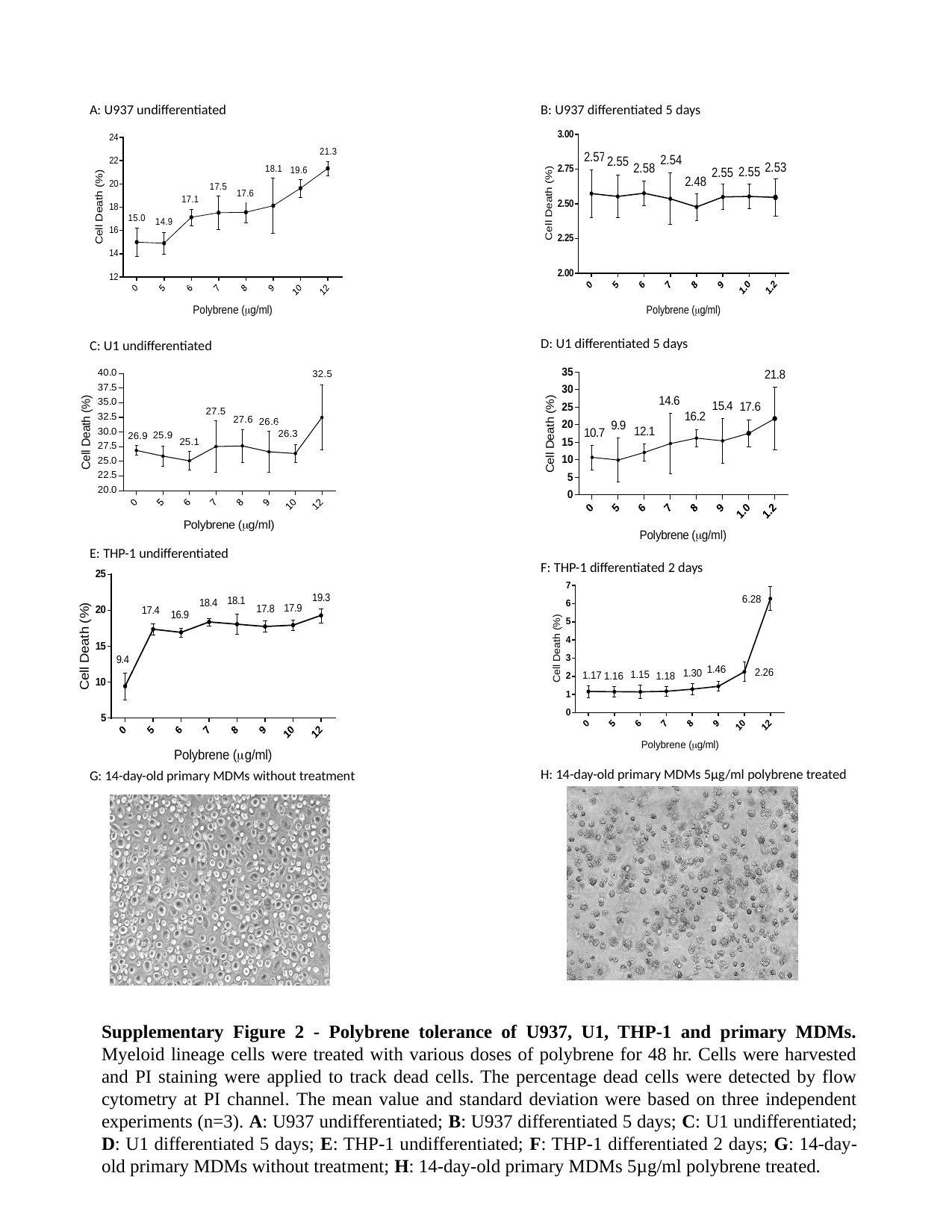

A: U937 undifferentiated
B: U937 differentiated 5 days
D: U1 differentiated 5 days
C: U1 undifferentiated
E: THP-1 undifferentiated
F: THP-1 differentiated 2 days
H: 14-day-old primary MDMs 5µg/ml polybrene treated
G: 14-day-old primary MDMs without treatment
Supplementary Figure 2 - Polybrene tolerance of U937, U1, THP-1 and primary MDMs. Myeloid lineage cells were treated with various doses of polybrene for 48 hr. Cells were harvested and PI staining were applied to track dead cells. The percentage dead cells were detected by flow cytometry at PI channel. The mean value and standard deviation were based on three independent experiments (n=3). A: U937 undifferentiated; B: U937 differentiated 5 days; C: U1 undifferentiated; D: U1 differentiated 5 days; E: THP-1 undifferentiated; F: THP-1 differentiated 2 days; G: 14-day-old primary MDMs without treatment; H: 14-day-old primary MDMs 5µg/ml polybrene treated.

## Slide 3
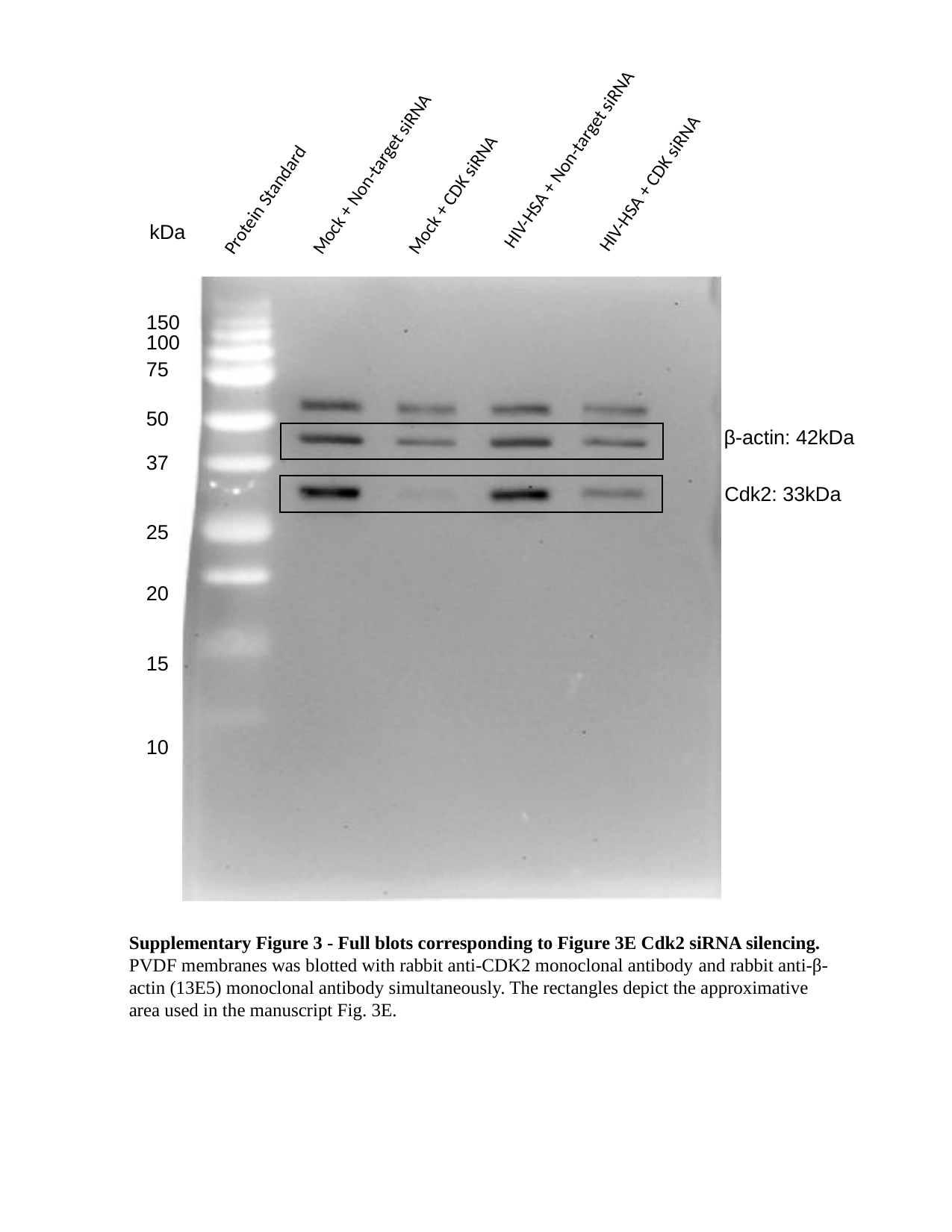

HIV-HSA + Non-target siRNA
HIV-HSA + CDK siRNA
Mock + CDK siRNA
Mock + Non-target siRNA
Protein Standard
kDa
150
100
75
50
37
25
20
15
10
β-actin: 42kDa
Cdk2: 33kDa
Supplementary Figure 3 - Full blots corresponding to Figure 3E Cdk2 siRNA silencing. PVDF membranes was blotted with rabbit anti-CDK2 monoclonal antibody and rabbit anti-β-actin (13E5) monoclonal antibody simultaneously. The rectangles depict the approximative area used in the manuscript Fig. 3E.

## Slide 4
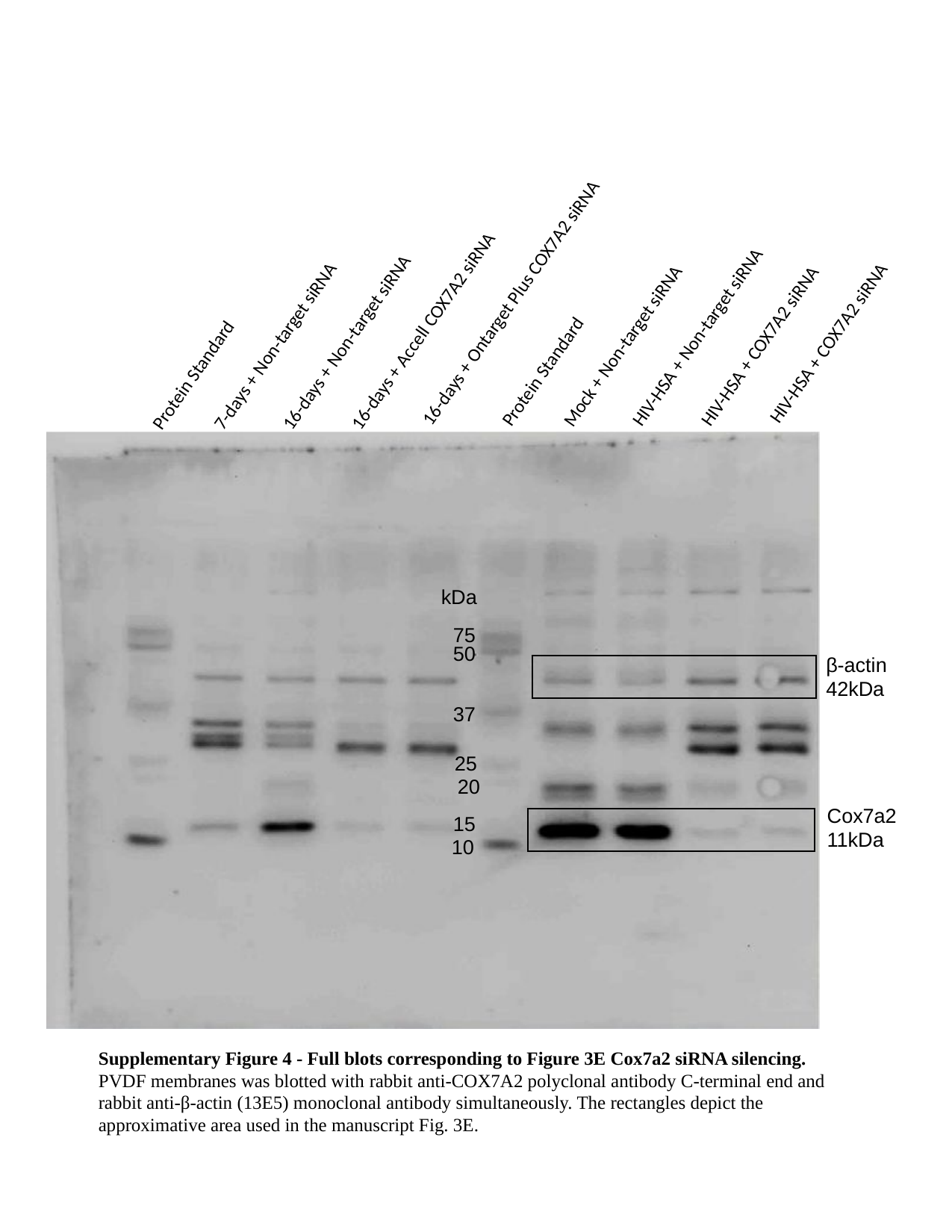

16-days + Ontarget Plus COX7A2 siRNA
16-days + Accell COX7A2 siRNA
16-days + Non-target siRNA
7-days + Non-target siRNA
Protein Standard
HIV-HSA + COX7A2 siRNA
HIV-HSA + COX7A2 siRNA
HIV-HSA + Non-target siRNA
Mock + Non-target siRNA
Protein Standard
kDa
75
50
37
25
20
15
10
β-actin
42kDa
Cox7a2
11kDa
Supplementary Figure 4 - Full blots corresponding to Figure 3E Cox7a2 siRNA silencing. PVDF membranes was blotted with rabbit anti-COX7A2 polyclonal antibody C-terminal end and rabbit anti-β-actin (13E5) monoclonal antibody simultaneously. The rectangles depict the approximative area used in the manuscript Fig. 3E.

## Slide 5
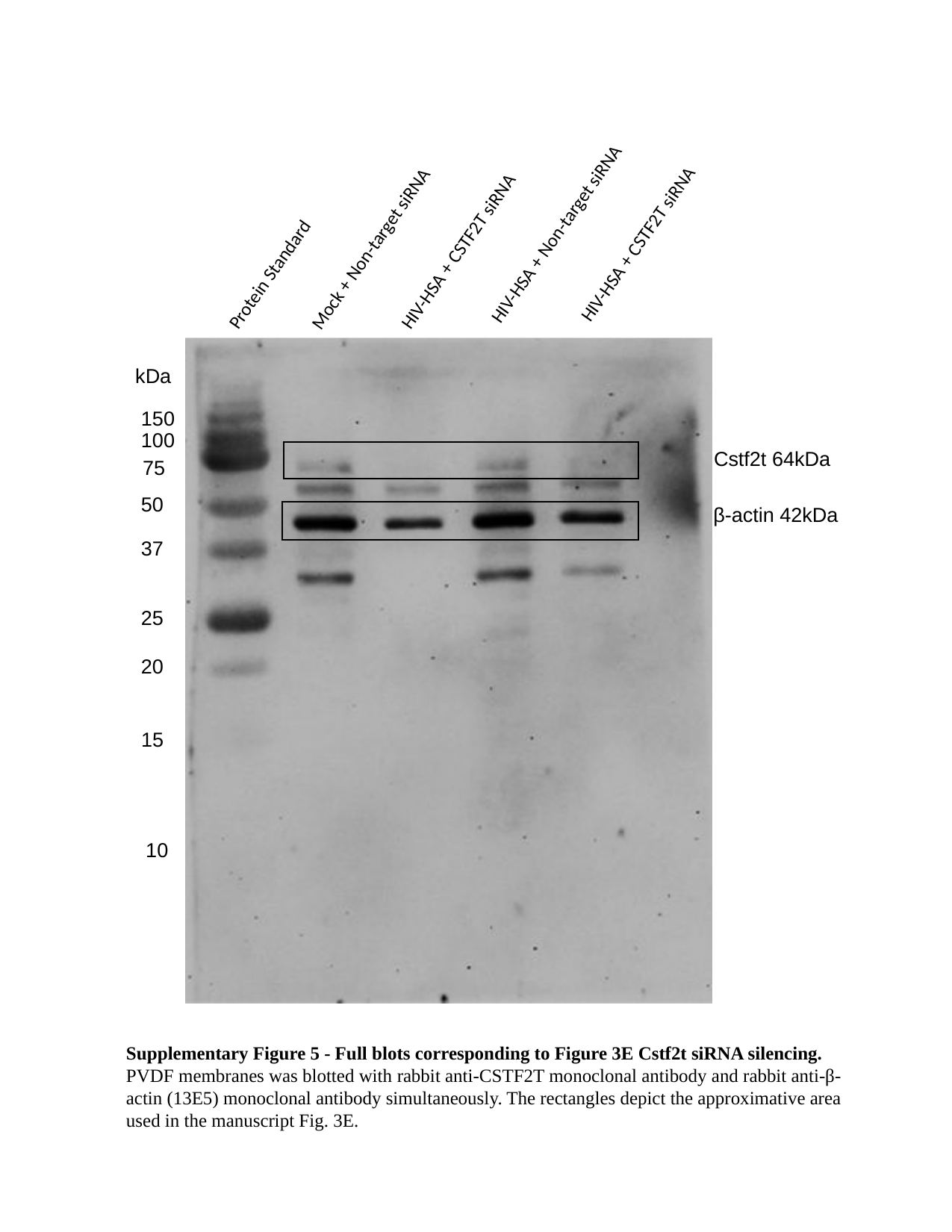

HIV-HSA + CSTF2T siRNA
HIV-HSA + Non-target siRNA
HIV-HSA + CSTF2T siRNA
Mock + Non-target siRNA
Protein Standard
kDa
150
100
75
50
37
25
20
15
10
Cstf2t 64kDa
β-actin 42kDa
Supplementary Figure 5 - Full blots corresponding to Figure 3E Cstf2t siRNA silencing. PVDF membranes was blotted with rabbit anti-CSTF2T monoclonal antibody and rabbit anti-β-actin (13E5) monoclonal antibody simultaneously. The rectangles depict the approximative area used in the manuscript Fig. 3E.

## Slide 6
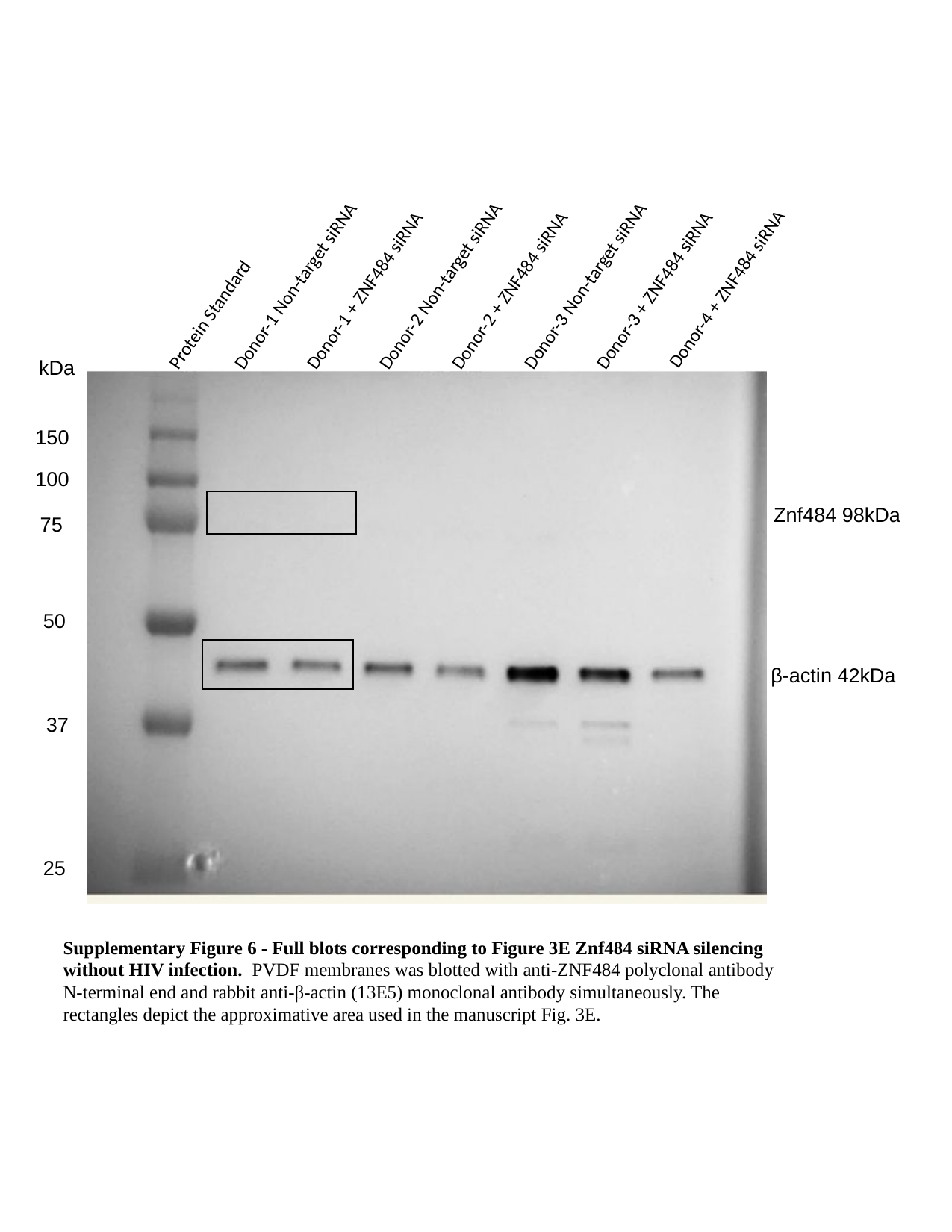

Donor-4 + ZNF484 siRNA
Donor-3 Non-target siRNA
Donor-3 + ZNF484 siRNA
Donor-1 Non-target siRNA
Donor-1 + ZNF484 siRNA
Donor-2 Non-target siRNA
Donor-2 + ZNF484 siRNA
Protein Standard
kDa
150
100
75
50
37
25
Znf484 98kDa
β-actin 42kDa
Supplementary Figure 6 - Full blots corresponding to Figure 3E Znf484 siRNA silencing without HIV infection. PVDF membranes was blotted with anti-ZNF484 polyclonal antibody N-terminal end and rabbit anti-β-actin (13E5) monoclonal antibody simultaneously. The rectangles depict the approximative area used in the manuscript Fig. 3E.

## Slide 7
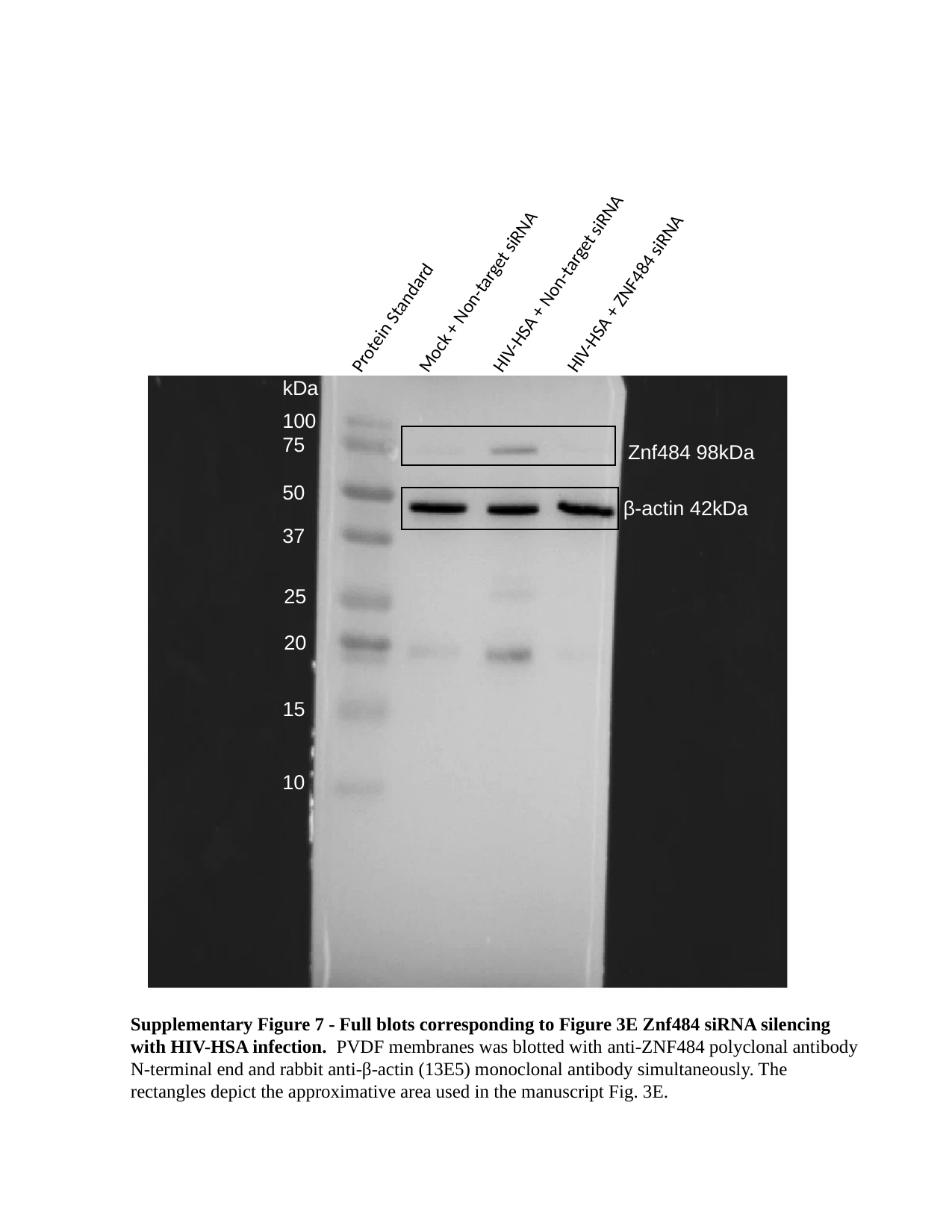

HIV-HSA + Non-target siRNA
Mock + Non-target siRNA
HIV-HSA + ZNF484 siRNA
Protein Standard
kDa
100
75
50
37
25
20
15
10
Znf484 98kDa
β-actin 42kDa
Supplementary Figure 7 - Full blots corresponding to Figure 3E Znf484 siRNA silencing with HIV-HSA infection. PVDF membranes was blotted with anti-ZNF484 polyclonal antibody N-terminal end and rabbit anti-β-actin (13E5) monoclonal antibody simultaneously. The rectangles depict the approximative area used in the manuscript Fig. 3E.

## Slide 8
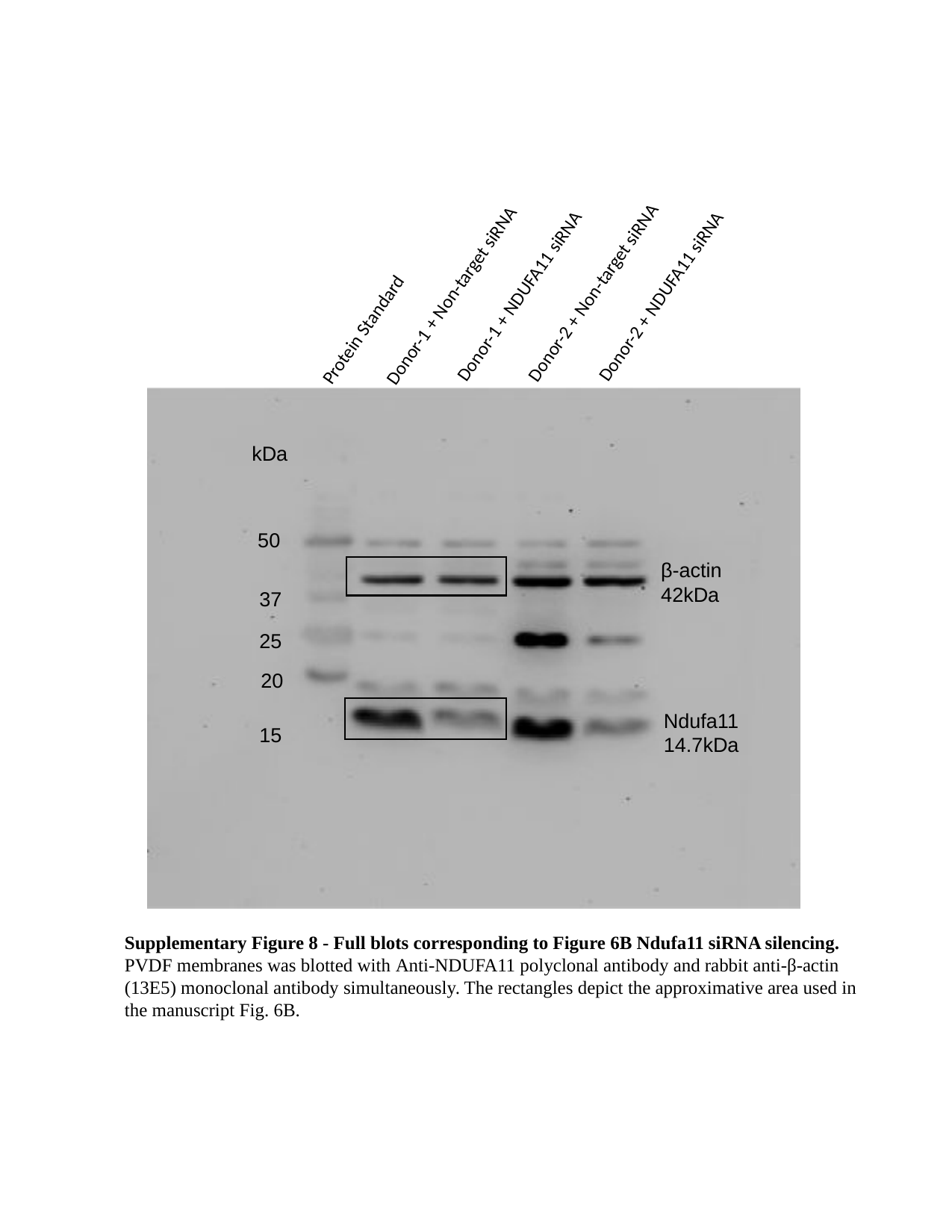

Donor-1 + NDUFA11 siRNA
Donor-2 + NDUFA11 siRNA
Donor-2 + Non-target siRNA
Donor-1 + Non-target siRNA
Protein Standard
kDa
50
37
25
20
15
β-actin
42kDa
Ndufa11
14.7kDa
Supplementary Figure 8 - Full blots corresponding to Figure 6B Ndufa11 siRNA silencing. PVDF membranes was blotted with Anti-NDUFA11 polyclonal antibody and rabbit anti-β-actin (13E5) monoclonal antibody simultaneously. The rectangles depict the approximative area used in the manuscript Fig. 6B.

## Slide 9
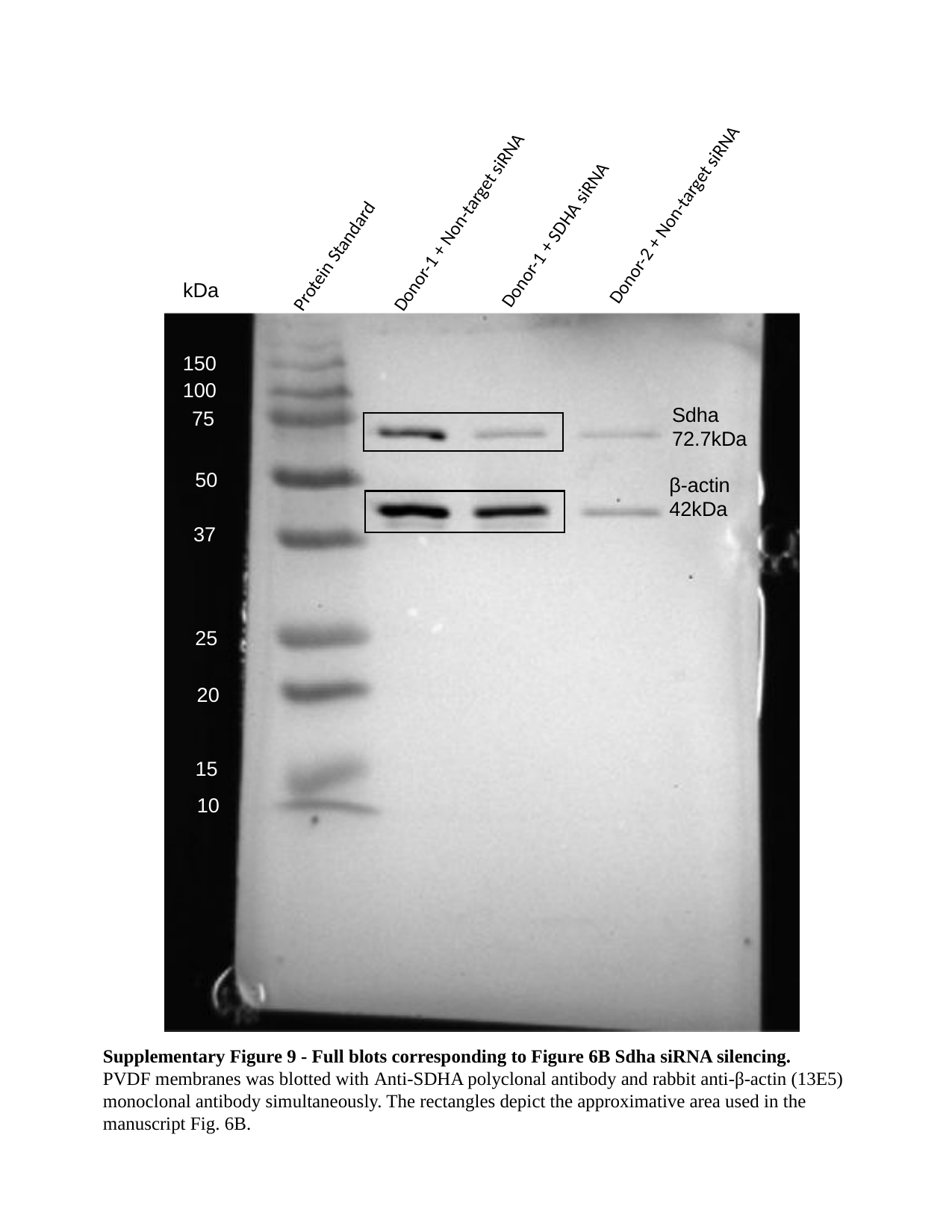

Donor-2 + Non-target siRNA
Donor-1 + SDHA siRNA
Donor-1 + Non-target siRNA
Protein Standard
kDa
150
100
75
50
37
25
20
15
10
Sdha
72.7kDa
β-actin
42kDa
Supplementary Figure 9 - Full blots corresponding to Figure 6B Sdha siRNA silencing. PVDF membranes was blotted with Anti-SDHA polyclonal antibody and rabbit anti-β-actin (13E5) monoclonal antibody simultaneously. The rectangles depict the approximative area used in the manuscript Fig. 6B.

## Slide 10
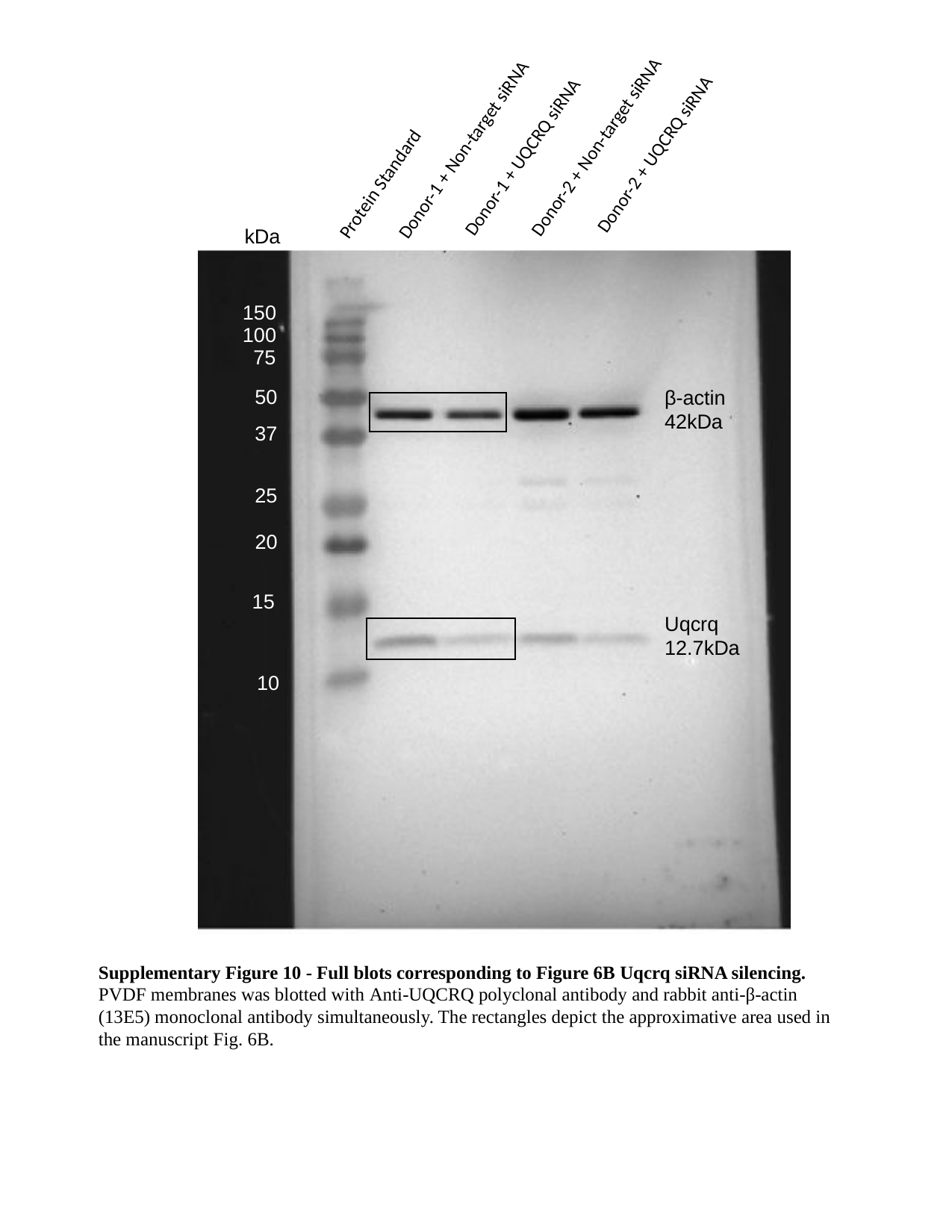

Donor-2 + UQCRQ siRNA
Donor-1 + UQCRQ siRNA
Donor-2 + Non-target siRNA
Donor-1 + Non-target siRNA
Protein Standard
kDa
150
100
75
50
37
25
20
15
10
β-actin
42kDa
Uqcrq
12.7kDa
Supplementary Figure 10 - Full blots corresponding to Figure 6B Uqcrq siRNA silencing. PVDF membranes was blotted with Anti-UQCRQ polyclonal antibody and rabbit anti-β-actin (13E5) monoclonal antibody simultaneously. The rectangles depict the approximative area used in the manuscript Fig. 6B.

## Slide 11
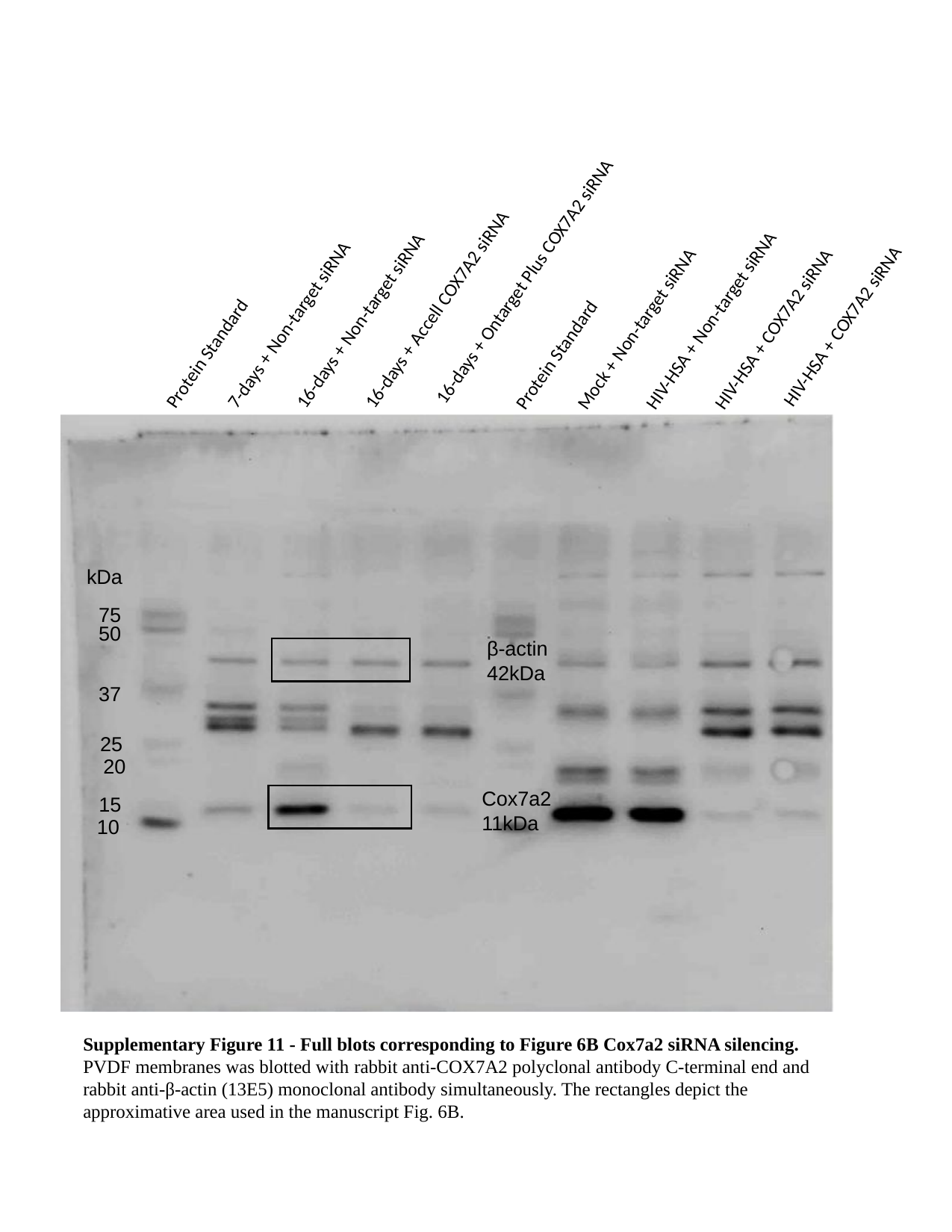

16-days + Ontarget Plus COX7A2 siRNA
16-days + Accell COX7A2 siRNA
16-days + Non-target siRNA
7-days + Non-target siRNA
Protein Standard
HIV-HSA + COX7A2 siRNA
HIV-HSA + COX7A2 siRNA
HIV-HSA + Non-target siRNA
Mock + Non-target siRNA
Protein Standard
kDa
75
50
37
25
20
15
10
β-actin
42kDa
Cox7a2
11kDa
Supplementary Figure 11 - Full blots corresponding to Figure 6B Cox7a2 siRNA silencing. PVDF membranes was blotted with rabbit anti-COX7A2 polyclonal antibody C-terminal end and rabbit anti-β-actin (13E5) monoclonal antibody simultaneously. The rectangles depict the approximative area used in the manuscript Fig. 6B.

## Slide 12
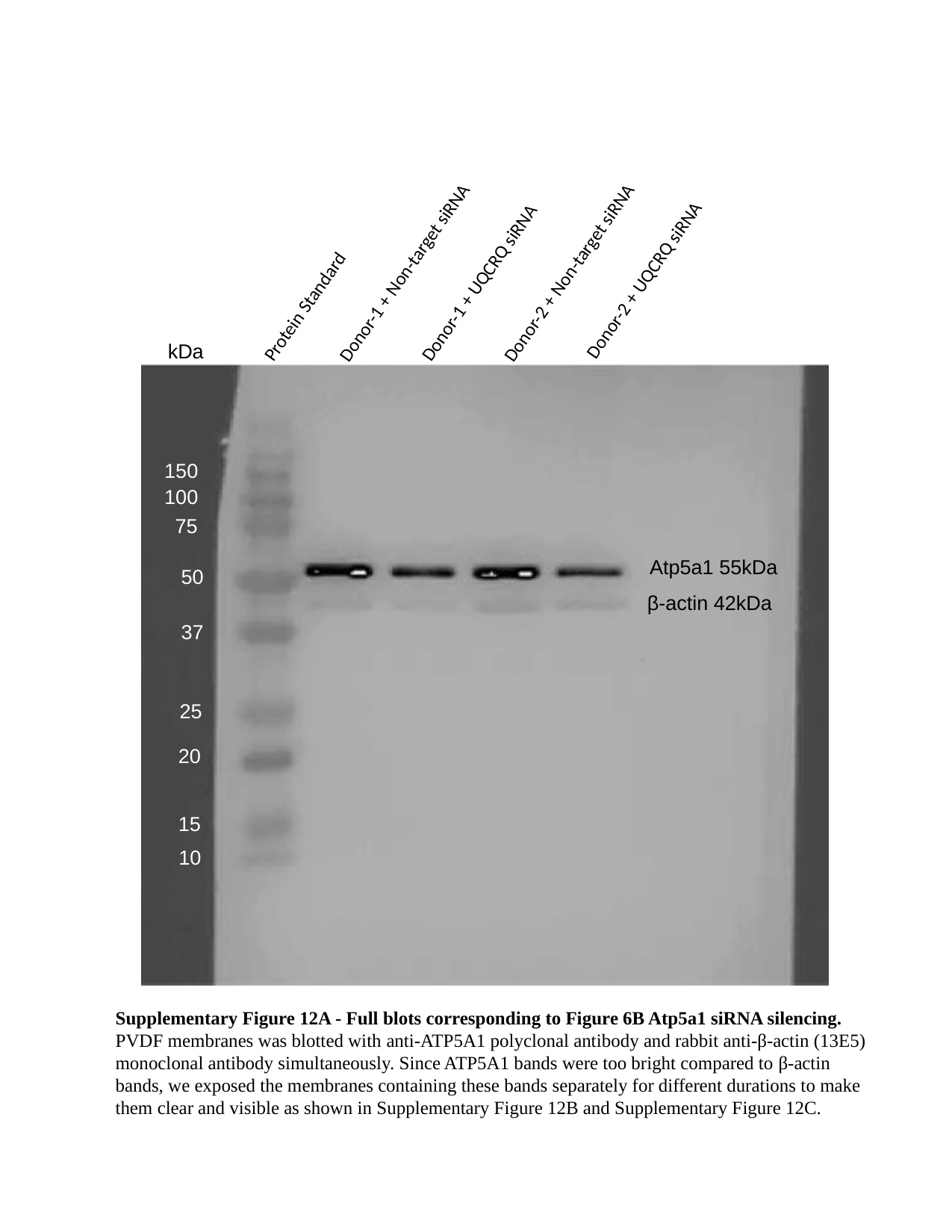

Donor-2 + UQCRQ siRNA
Donor-1 + UQCRQ siRNA
Donor-1 + Non-target siRNA
Donor-2 + Non-target siRNA
Protein Standard
kDa
150
100
75
50
37
25
20
15
10
Atp5a1 55kDa
β-actin 42kDa
Supplementary Figure 12A - Full blots corresponding to Figure 6B Atp5a1 siRNA silencing. PVDF membranes was blotted with anti-ATP5A1 polyclonal antibody and rabbit anti-β-actin (13E5) monoclonal antibody simultaneously. Since ATP5A1 bands were too bright compared to β-actin bands, we exposed the membranes containing these bands separately for different durations to make them clear and visible as shown in Supplementary Figure 12B and Supplementary Figure 12C.

## Slide 13
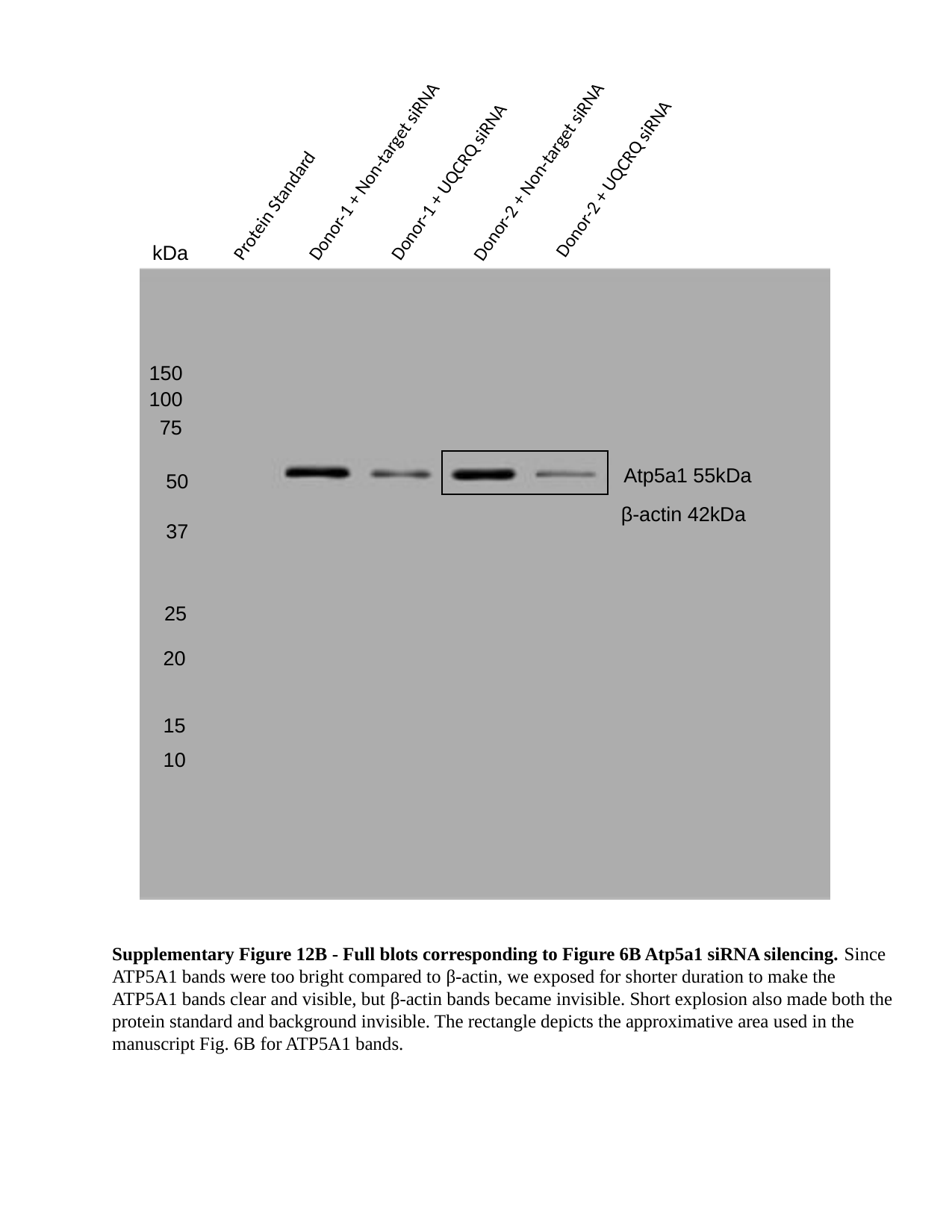

Donor-2 + UQCRQ siRNA
Donor-1 + UQCRQ siRNA
Donor-1 + Non-target siRNA
Donor-2 + Non-target siRNA
Protein Standard
kDa
150
100
75
50
37
25
20
15
10
Atp5a1 55kDa
β-actin 42kDa
Supplementary Figure 12B - Full blots corresponding to Figure 6B Atp5a1 siRNA silencing. Since ATP5A1 bands were too bright compared to β-actin, we exposed for shorter duration to make the ATP5A1 bands clear and visible, but β-actin bands became invisible. Short explosion also made both the protein standard and background invisible. The rectangle depicts the approximative area used in the manuscript Fig. 6B for ATP5A1 bands.

## Slide 14
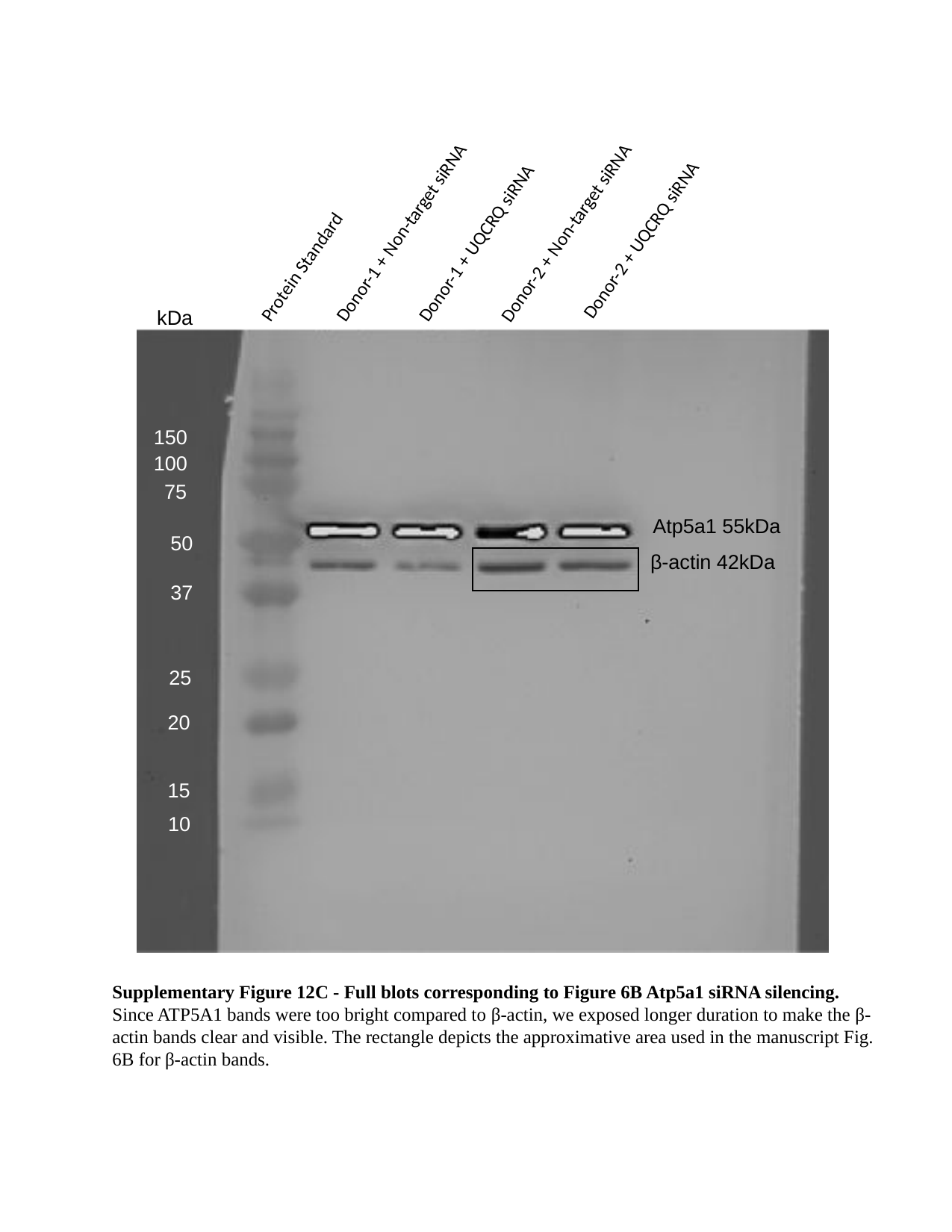

Donor-2 + UQCRQ siRNA
Donor-1 + UQCRQ siRNA
Donor-1 + Non-target siRNA
Donor-2 + Non-target siRNA
Protein Standard
kDa
150
100
75
50
37
25
20
15
10
Atp5a1 55kDa
β-actin 42kDa
Supplementary Figure 12C - Full blots corresponding to Figure 6B Atp5a1 siRNA silencing. Since ATP5A1 bands were too bright compared to β-actin, we exposed longer duration to make the β-actin bands clear and visible. The rectangle depicts the approximative area used in the manuscript Fig. 6B for β-actin bands.
